# Micro/Nanostructured Materials and Microfluidic Diagnostic Platforms Enable Multimodal Detection of Circulating Tumor Cells

**DOI:** 10.1002/exp2.70239

**Published:** 2026-10-11

**Authors:** Hao Li, Jiaojiao Sun, Kunguo Liu, Shijie Wang, Bowen Fu, Hong Zhu, Peng Cao, Xin Han

**Affiliations:** ^1^ Department of Medical Oncology the First Affiliated Hospital of Soochow University Suzhou China; ^2^ State Key Laboratory on Technologies for Chinese Medicine Pharmaceutical Process Control and Intelligent Manufacture Nanjing University of Chinese Medicine Nanjing China; ^3^ Suzhou Institute of Biomedical Engineering and Technology Chinese Academy of Sciences Suzhou China; ^4^ School of Electrical and Electronic Engineering Nanyang Technological University Singapore Singapore

**Keywords:** circulating tumor cells, efficient enrichment, micro/nanostructured materials, microfluidic technology, multidimensional molecular profiling

## Abstract

Circulating tumor cells (CTCs), as pivotal targets in liquid biopsy, offer dynamic, multidimensional insights into tumor heterogeneity and metastatic progression. However, their extreme rarity, pronounced heterogeneity, and the complexity of the blood microenvironment severely constrain detection accuracy. Advances in micro/nanomaterials and functional device engineering have enabled substantial improvements in CTC enrichment efficiency by refining the topographical and chemical properties of capture interfaces. These innovations also provide a compatible foundation for multimodal molecular profiling. This review systematically outlines the structure–function relationships of functionalized nanomaterials, microfiltration substrates, and biomimetic microfluidic devices, highlighting how material parameters—such as nanoscale architectures and membrane pore sizes—govern CTC capture performance. We further explore the integration of micro/nanomaterials with functional platforms for multidimensional molecular analyses across DNA, RNA, and protein levels. Coupled with emerging digital PCR technologies, these systems show promise in overcoming the sensitivity barriers associated with rare mutation detection and protein quantification, enabling multi‐omics data fusion. We also discuss how artificial intelligence (AI)‐driven feature selection, clustering, and prediction integrate multidimensional data to decode tumor heterogeneity and guide disease monitoring. Despite rapid progress, key challenges remain—including the lack of standardization, limited integration of multi‐omic datasets, and the need for robust clinical validation. Future directions may lie in the convergence of microfluidic‐integrated materials, single‐cell multi‐omics, and AI‐driven analytics to establish dynamic, high‐resolution monitoring platforms that bridge the gap between fundamental research and clinical precision oncology.

## Graphical abstract

Engineered micro/nanomaterials enable high‐efficiency CTC capture, release, and multimodal molecular profiling. Integrated with AI‐driven analytics, these platforms decode tumor heterogeneity and metastatic dynamics, advancing real‐time monitoring and precision oncology through DNA, RNA, and protein cross‐scale data fusion.

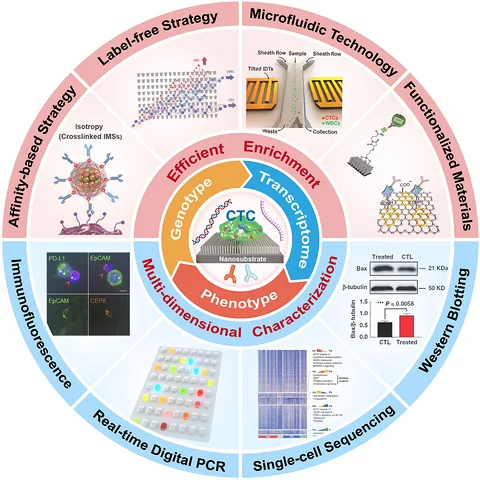

## 1 Introduction

Cancer remains a major global health burden, primarily due to late‐stage diagnosis and limited therapeutic efficacy [1]. Its high metastatic potential further complicates disease management and outcomes [2]. Despite advances in diagnostic imaging and molecular profiling, routine clinical practices—such as periodic liver ultrasound with or without AFP testing in hepatocellular carcinoma—often fall short in meeting the multifaceted needs of cancer diagnosis and monitoring [3]. Traditional diagnostic approaches suffer from low sensitivity and provide limited molecular insights into tumor origin, failing to capture the full complexity and dynamic nature of tumor biology. These limitations underscore the urgent need for novel, highly sensitive platforms capable of providing comprehensive insights into tumor heterogeneity, therapeutic response, and disease progression.

In this context, liquid biopsy technologies powered by micro/nanomaterials and functional devices are emerging as transformative tools for cancer biomarkers detection [4]. Key analytes include extracellular vesicles (EVs) [5], circulating tumor DNA (ctDNA) [6], and particularly, circulating tumor cells (CTCs) [7]. As “seeds” of metastasis and a dynamic “liquid window” into the molecular traits of both primary and metastatic lesions, CTCs offer real‐time access to tumor‐derived DNA, RNA, and proteins [8, 9, 10]. However, the rarity of CTCs (typically one per 10^5^–10^6^ blood cells) and their phenotypic plasticity—particularly during epithelial–mesenchymal transition (EMT)—pose significant challenges for both detection sensitivity and specificity [11, 12, 13]. Current enrichment strategies exploit biophysical properties [14], immunoaffinity [15], or hybrid approaches [16], yet their clinical translation is hindered by issues such as nonspecific adsorption on material surfaces, insufficient integration of multi‐omic data, and a lack of standardized protocols for dynamic monitoring. Addressing these barriers is critical to realizing the full clinical potential of CTC‐based diagnostics.

Innovations in microfluidic cross‐linked materials have increasingly taken center stage in advancing CTC detection. Engineered microporous structures—such as windmill‐like pore arrays—combined with anti‐fouling polymer surface modifications, significantly improve both the capture efficiency and purity of CTCs [17]. Antibody‐ or aptamer‐functionalized nanostructured materials, optimized for ligand density and spatial configuration, enhance the selective recognition of heterogeneous CTC subpopulations, including those with low EpCAM expression [18, 19]. Meanwhile, biomimetic microfluidic chips that replicate biological surface topographies—such as octopus‐tentacle‐inspired micropillar arrays—enable efficient and selective CTC capture and release [20]. These advances in micro/nanomaterials and functional microdevices not only provide compatible platforms for multimodal molecular profiling techniques, including digital PCR and mass spectrometry, but also lay the foundation for integrated multi‐omic analyses of CTC genomes, transcriptomes, and proteomes—critical for dissecting tumor heterogeneity and guiding precision oncology.

This review centers on the convergence of materials science and biomedicine, a synergy that is driving advances not only in diagnostic platforms but also in innovative therapeutic modalities [21, 22]. We first outline the origins, evolution, and clinical relevance of liquid biopsy technologies, then highlight CTCs as a pivotal focus within this domain. We systematically examine the structure–function relationships of micro/nanomaterial‐based platforms for efficient CTC enrichment, and discuss strategies for integrating multidimensional molecular detection—such as next‐generation PCR for cross‐scale protein‐RNA quantification—built upon microfluidic cross‐linked materials. Finally, we explore the promise of artificial intelligence (AI) in decoding CTC‐derived multi‐omic datasets. Through a tightly integrated materials–device–algorithm framework, micro/nanomaterials and functional microdevices offer high‐sensitivity, multidimensional, and clinically scalable solutions for building real‐time tumor monitoring networks, paving the way toward a transformative paradigm in longitudinal cancer management.

## 2 Overview of Liquid Biopsy

### 2.1 The Evolution of Liquid Biopsy

Liquid biopsy has emerged as a transformative approach for cancer detection and monitoring, offering a minimally invasive means to access tumor‐derived information [23]. The concept dates back to the late 1860s, when Australian physician Thomas Ashworth first described CTCs in the bloodstream [24]. Subsequent advances in immunofluorescence and flow cytometry significantly improved the sensitivity and specificity of CTC detection [25], fueling a surge in basic research that established the link between CTCs and cancer metastasis [26]. In 2004, the CellSearch system became the first CTC detection platform approved by the U.S. Food and Drug Administration (FDA), marking the clinical debut of CTC‐based diagnostics [12].

The study of EVs, particularly exosomes, began in the 1980s, when small vesicles were observed to form and be released from mammalian cells [27]. Initially regarded as cellular waste carriers, exosomes were redefined in 1996 as critical mediators of intercellular communication [28]. By 2007, they were shown to carry tumor‐associated proteins and RNAs, actively shaping the tumor microenvironment [29]. Since then, exosomes have gained considerable attention for their roles in biomarker discovery, intercellular signaling, and therapeutic applications [30].

Research on ctDNA began in 1948 with the discovery of extracellular DNA in human blood [31]. In 1977, elevated levels of free DNA in the serum of cancer patients were reported, providing a foundation for ctDNA analysis [32]. The clinical utility of ctDNA expanded significantly by 2014, when it began to be routinely used for monitoring treatment response and detecting cancer recurrence [33].

From the initial observation of CTCs to the extensive study of exosomes and ctDNA, the development of liquid biopsy has spanned decades of scientific inquiry, technological innovation, and clinical translation. Today, it offers unprecedented opportunities for real‐time tumor profiling, prognosis evaluation, and therapeutic monitoring across a broad spectrum of malignancies.

### 2.2 Comprehensive Molecular Insights Through Liquid Biopsy in Precision Oncology

In the context of precision oncology, a comprehensive understanding of tumor molecular profiles is essential to reveal the underlying diversity and complexity of cancer [34]. Currently, conventional molecular analysis still relies predominantly on tissue biopsies or surgical specimens. By directly sampling tumor sites, tissue biopsy enables morphological and structural assessment to guide staging, diagnosis, and treatment planning [34]. However, this approach presents limitations, including challenges in tumor accessibility, limited sample quantity and quality, and the inability to capture temporal and spatial tumor heterogeneity. As a result, it often provides only a static snapshot of a dynamic disease process.

Liquid biopsy has emerged as a minimally invasive alternative, offering access to real‐time, system‐wide tumor information. Because blood circulates through most tumor sites, particularly in advanced cancers, blood‐based sampling may more accurately reflect tumor heterogeneity than small tissue biopsies [11]. By analyzing circulating tumor components such as CTCs, ctDNA, and exosomes, liquid biopsy provides a dynamic molecular landscape of cancer, enabling longitudinal monitoring of disease evolution and therapeutic response [35].

The logistical and biological constraints of tissue sampling have intensified interest in liquid biopsy. Among its most established analytes are ctDNA and CTCs [36]. In ctDNA, somatic mutations and copy number variations (CNVs) can be detected via droplet digital PCR (ddPCR) or next‐generation sequencing (NGS), and have been widely adopted to predict treatment response and assess therapeutic efficacy [11]. For example, EGFR mutation testing guides the use of tyrosine kinase inhibitors such as gefitinib and erlotinib in non‐small cell lung cancer (NSCLC) [37]. Recently, ctDNA methylation analysis has gained traction as a complementary tool to mutation and CNV profiling, offering clues to tumor origin in cancers of unknown primary [38]. Longitudinal monitoring of ctDNA mutations—such as PIK3CA and ESR1 in breast cancer—can reveal emerging resistance mechanisms [39] and track minimal residual disease (MRD) following curative treatment, enabling early relapse detection [40].

CTCs, tumor cells shed into the bloodstream from the primary lesion, present greater technical challenges in enrichment and detection, but offer expanded possibilities for biomarker discovery. Protein expression analysis of CTCs, such as PD‐L1 [41] or ARV7 [42], can inform immunotherapy or androgen receptor‐targeted treatment decisions in cancers like lung and prostate, respectively. Genomic analysis at the single‐cell level has unveiled intrapatient CTC heterogeneity and clonal evolution [43], while serial CTC enumeration has been used to monitor treatment response—for instance, CTC decline correlates with therapeutic benefit in breast cancer [12]. In some cases, CTCs have been used to generate patient‐derived xenograft (CDX) models in immunodeficient mice, supporting preclinical drug development [44]. Ongoing efforts to culture CTCs ex vivo may eventually enable real‐time drug sensitivity testing to support individualized treatment [13].

Despite its promise, liquid biopsy still faces significant limitations. Chief among them is the lack of standardized protocols, which hampers cross‐comparison between studies and leads to methodological variability. Moreover, while liquid biopsy excels at detecting specific mutations or profiling genomic and transcriptomic data, it often falls short in integrating multidimensional molecular information. Its sensitivity and specificity, particularly for early cancer detection, remain suboptimal due to the low abundance of ctDNA and CTCs in circulation.

Future advances in liquid biopsy will focus on several fronts. Standardized detection platforms are urgently needed to harmonize results across different biomarkers. Deeper understanding of tumor biology will enable integration of diverse molecular data—combining ctDNA, CTCs, and exosomes, or analyzing multiple molecular layers (DNA, RNA, protein) within a single analyte—to construct a more holistic tumor profile. As technologies mature, liquid biopsy is poised to drive the next wave of precision oncology, reshaping cancer diagnostics and treatment monitoring.

## 3 Overview of CTCs

CTCs represent a critical component of liquid biopsy, defined as cells shed from solid tumors into the bloodstream. They are distinct from hematological malignancies and act as cellular “seeds” with the potential to initiate distant metastases [45, 46, 47]. Unlike traditional cancer biomarkers, CTCs offer a dynamic and multidimensional snapshot of the living tumor within the patient, encompassing tumor‐derived DNA, mRNA, and protein signatures [8, 9]. This unique molecular richness renders CTCs invaluable for elucidating tumor progression, assessing therapeutic response, and ultimately improving patient outcomes. However, it is crucial to note that not all detected CTCs are viable or metastatic. The CTC population is heterogeneous, comprising apoptotic cells, transient bystanders with limited survival capacity, and a smaller subset of viable cells possessing metastatic potential. It is this latter subpopulation that holds the greatest clinical significance for understanding metastasis and treatment resistance.

The clinical importance of CTCs lies not only in their prognostic and predictive potential, but also in their ability to uncover the underlying mechanisms of cancer metastasis, drug resistance, and tumor evolution. Given the functional heterogeneity of CTCs, a key advancement in the field is the move beyond simple enumeration towards the identification and molecular characterization of specific subpopulations—such as those undergoing EMT, possessing stem‐cell‐like properties, or forming clusters—which are more strongly associated with metastatic progression and poor outcomes. This section outlines the biological background and clinical significance of CTCs, highlighting their role as a bridge between tumor biology and precision oncology.

### 3.1 The Link Between CTCs and Metastasis

The initial observation of CTCs dates back to the early 19th century, when Thomas Ashworth reported the presence of epithelial‐like cells in the blood of a deceased cancer patient [9]. He hypothesized that these cells originated from the primary tumor, suggesting a potential role in metastasis. Subsequent studies over the following decades consistently detected tumor cells in the blood of patients with advanced malignancies—sometimes even before death—laying the conceptual foundation for CTCs as “seeds” of micrometastases [48].

The recognition of CTCs as metastatic precursors emerged through more than a century of evolving insight. In 1889, Stephen Paget proposed the seminal “seed and soil” hypothesis, suggesting that cancer cells disseminate through the bloodstream and grow only in favorable environments within distant organs [49]. Like seeds that germinate only in suitable soil, tumor cells require permissive microenvironments to establish secondary lesions. Although initially met with skepticism, the model gained acceptance as empirical evidence accumulated throughout the mid‐20th century, confirming that cells from primary tumors can indeed disseminate hematogenously to colonize distant sites [50].

In 1959, a 5‐year follow‐up study of 125 cancer patients provided one of the first longitudinal investigations into CTCs, tracking their presence before and after surgery [8, 51, 52]. This spurred additional studies examining perioperative CTC dynamics. Later, landmark experiments by Fidler and Kripke, involving intravenous injection of malignant melanoma cells into mice, demonstrated that metastasis is driven by pre‐existing variant clones within the primary tumor. Their findings highlighted the role of tumor heterogeneity and clonal selection in metastatic progression [46].

Today, it is well established that CTCs detach from primary tumors and enter the circulatory system through a multistep process. Analogous to seeds that eventually sprout in favorable soil, these cells can colonize distant tissues, forming metastatic lesions and completing the cascade of cancer dissemination.

### 3.2 Biological Characteristics of CTCs

Although extremely rare, CTCs play a pivotal role in cancer metastasis. These cells exhibit complex and diverse biological characteristics, most notably significant molecular and phenotypic heterogeneity driven by transitions between EMT and mesenchymal–epithelial transition (MET) [53]. Figure 1 illustrates this metastatic journey. EMT enables epithelial tumor cells to lose intercellular adhesion and acquire mesenchymal, invasive properties [54]. Through EMT activation, tumor cells detach from the basement membrane, enter the circulatory system as CTCs, and disseminate to distant sites. Upon reaching secondary organs, these cells undergo MET, reverting to an epithelial phenotype and proliferating to establish macrometastases [55].

**FIGURE 1:**
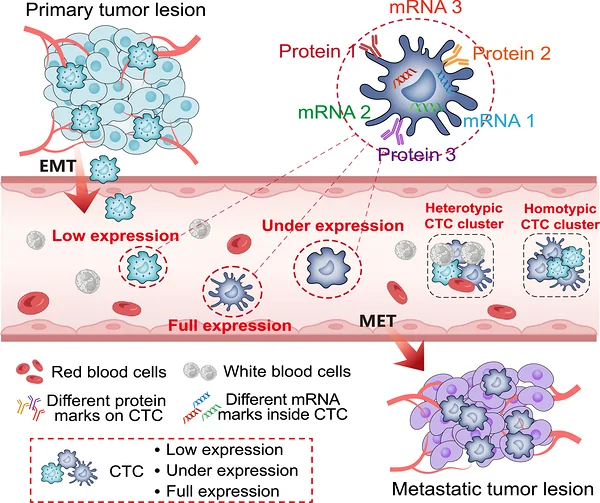
Biological characteristics of CTCs during metastasis. CTCs exhibit marked molecular and phenotypic heterogeneity throughout EMT and MET, displaying diverse protein expression patterns—ranging from full to partial or low expression. Within the bloodstream, CTCs interact dynamically with various cellular components of the microenvironment, forming homotypic or heterotypic clusters. These clusters undergo continuous changes in intracellular RNA profiles and surface protein expression, reflecting the adaptive strategies of CTCs during metastatic progression. Adapted under the terms of the Creative Commons CC BY license.[56] Copyright 2024, The Author(s).

The metastatic cascade thus hinges on a delicate balance between EMT and MET states. As shown in Figure 1, CTCs modulate gene expression and phenotypic features in response to their microenvironment and survival demands. This results in three characteristic protein‐to‐mRNA expression patterns: low expression, full expression, and under expression of transition‐related markers [56]. For instance, CTCs may fully express EMT markers during detachment and intravasation to enhance invasiveness [47]. In transitional phases, they often partially express both epithelial and mesenchymal markers, enabling adaptability and intermediate metastatic competency [45]. In other contexts—such as immune evasion during colonization—they may suppress EMT markers altogether [57]. The plasticity of gene expression and dynamic interconversion between EMT and MET states allow CTCs to survive in circulation and successfully establish metastatic lesions. These phenotypic shifts not only dictate their migratory and invasive capabilities but also influence their ability to proliferate and colonize distant tissues [45, 47, 54, 55, 57].

Beyond intrinsic features, CTCs engage in complex interactions with various bloodborne cells. An estimated 1×10^6^ tumor cells per gram of tumor are released into circulation, equating to approximately one CTC per 10^5^‐10^6^ peripheral blood mononuclear cells [58]. However, the vast majority of CTCs are eliminated by hemodynamic forces or immune surveillance [59]. A select few, however, form multicellular clusters—CTC clusters—with significantly enhanced metastatic potential [60]. CTC clusters, defined as aggregates of two or more tumor cells with intact nuclei, exhibit brief circulation times: approximately 25–30 min for single CTCs and just 6–10 min for clusters, likely due to rapid lodging in capillary beds at secondary sites [61, 62].

Despite their transient presence, CTC clusters show greater metastatic efficiency than solitary CTCs. They also display elevated levels of EMT and stemness markers [59, 61]. As shown in Figure 1, CTC clusters are classified as either homotypic or heterotypic. Homotypic clusters form through adhesion molecules such as CD44, E‐cadherin, desmoplakin, and ICAM1, facilitating intercellular stability, downstream signaling, and enhanced invasiveness [63, 64, 65]. A higher count of single CTCs is associated with poorer survival, a trend even more pronounced with abundant CTC clusters [66]. Heterotypic clusters, by contrast, consist of CTCs bound to other blood‐derived cells—such as leukocytes, neutrophils, fibroblasts, platelets, or myeloid‐derived suppressor cells [67, 68, 69]. Patients harboring CTC–leukocyte clusters exhibit worse overall survival compared to those without [70]. CTCs in association with neutrophils or fibroblasts demonstrate greater proliferative, invasive, and migratory activity [69]. Platelet‐associated CTCs are believed to evade immune detection more effectively [71].

Together, these dynamic molecular and phenotypic changes during EMT–MET transitions and the formation of multicellular aggregates underscore the multifaceted biology of CTCs. Their rarity, heterogeneity, and interactions with the circulating microenvironment pose significant challenges for detection, yet also offer valuable insights into the mechanisms of metastasis and new avenues for therapeutic intervention.

### 3.3 Clinical Significance of CTCs

CTCs hold substantial value in both cancer research and clinical practice, offering critical insights into tumor biology and informing patient management. As shown in Figure 2, the clinical utility of CTCs in hepatocellular carcinoma (HCC) spans multiple domains, including companion diagnostics, prognostic assessment, disease progression monitoring, and personalized therapy. These applications highlight the translational potential of CTC‐based analyses to refine cancer detection, guide treatment decisions, and improve clinical outcomes.

**FIGURE 2:**
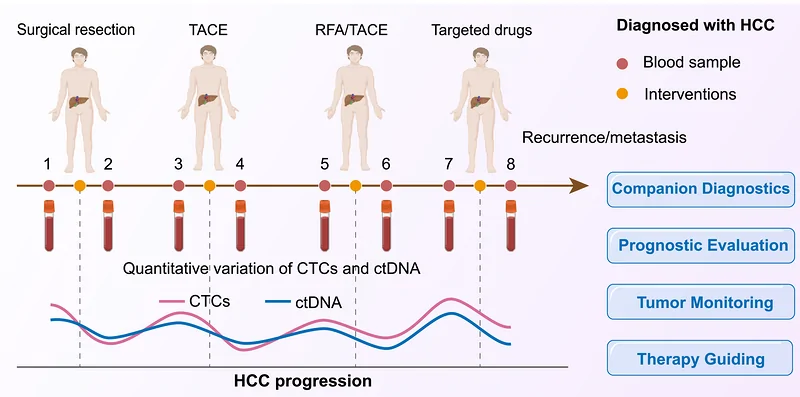
Clinical applications of CTCs and ctDNA in patients with HCC. Liquid biopsy enables real‐time monitoring of treatment response and disease recurrence in HCC. Following initial diagnosis, sequential blood sampling was performed at defined clinical stages—before and after surgery, TACE, RFA/TACE, and targeted therapy (red dots indicate sampling points; orange dots, intervention time points). The pink and blue curves below illustrate the relative quantitative dynamics of CTCs and ctDNA, respectively, serving as biomarkers for companion diagnosis, prognosis assessment, tumor surveillance, and therapeutic guidance. The ctDNA signal (blue) largely parallels tumor burden: levels decline after surgical or interventional procedures, exhibit transient “rebound peaks” during TACE/RFA due to tumor necrosis, then decrease again; progressive elevation indicates residual disease, drug resistance, or recurrence. The CTC profile (pink) follows a similar trend but fluctuates more markedly, influenced by procedural disturbance and phenotypic invasiveness—showing transient postoperative spikes before decline, and sharper increases upon disease progression or metastasis. Together, ctDNA and CTC offer complementary insights: ctDNA quantitatively reflects tumor‐derived nucleic acid burden, while CTCs provide cellular‐level information on phenotype and metastatic potential. Their integrated interpretation enhances the reliability and robustness of therapeutic response evaluation and recurrence detection. The curves shown are schematic, representing population‐level trends rather than absolute quantitative values or individualized trajectories. TACE: transarterial chemoembolization; RFA: radiofrequency ablation.

The presence of CTCs in the bloodstream offers valuable prognostic insights and [72], when combined with ctDNA or other diagnostic modalities, can serve as a companion or adjunctive diagnostic tool in cancer care. In HCC, EpCAM‐positive CTCs have been extensively studied [73]. Evidence indicates that patients with EpCAM^+^ CTCs exhibit significantly shorter overall survival (OS) and disease‐free survival (DFS), and are often associated with more aggressive clinical features [74]. Although the precise threshold for defining CTC positivity remains under debate, a higher CTC count generally correlates with poorer prognosis. However, only approximately 35% of HCC cases express EpCAM, limiting the utility of CTCs as a standalone diagnostic or early screening tool in this context [75]. To enhance predictive accuracy for recurrence and prognosis, several studies have explored alternative CTC markers or marker combinations. For instance, the coexistence of EpCAM^+^ CTCs with regulatory T cells (Tregs) marked by CD4^+^, CD25^+^, and Foxp3^+^ has been linked to HCC recurrence [76]. Patients with high CTC/Treg levels showed a markedly elevated risk of recurrence following surgery compared to those with low levels (66.7% vs. 10.3%, *p* < 0.001) [76]. In addition, other CTC subtypes have shown promise for early recurrence prediction [77]. A study involving 62 HCC patients found significantly higher levels of mesenchymal and hybrid CTCs in those who experienced recurrence. Mesenchymal CTC positivity (HR = 3.453, *p* = 0.007) emerged as an independent risk factor for early relapse [77]. Collectively, these findings suggest that diverse CTC phenotypes and marker combinations can serve as valuable indicators for HCC recurrence and prognosis. Nevertheless, the lack of HCC‐specificity in many of these markers continues to limit their utility in early detection.

CTCs obtained through liquid biopsy display considerable genetic and phenotypic diversity, mirroring the intratumoral heterogeneity of the primary tumor [78]. Numerous studies have identified distinct CTC subpopulations. In patients with pancreatic ductal adenocarcinoma (PDAC), for instance, two major CTC subsets have been described—one expressing the epithelial cell adhesion molecule (CTC^EpCAM^) and the other expressing fibroblast activation protein alpha (CTC^FAPα^). The ratio of CTC^FAPα^ to CTC^EpCAM^ was shown to distinguish responders from non‐responders to the PARP inhibitor niraparib [79]. In non‐small cell lung cancer (NSCLC), distinct CTC gene expression profiles have been associated with treatment resistance (e.g., MET and HER3) and metastatic potential (e.g., ALDH1) [80]. Tumor heterogeneity has long been recognized as a key factor in cancer's ability to adapt to microenvironmental stress, with distinct subclones evolving to enhance invasiveness or evade therapeutic pressure. The study of CTC heterogeneity thus provides a powerful means to monitor tumor dynamics and uncover actionable features of tumor progression. By characterizing CTC subtypes and their evolving traits, clinicians may better tailor treatment strategies, ultimately advancing the goals of personalized cancer therapy.

Changes in CTC count and molecular characteristics during treatment can serve as indicators of therapeutic response or emerging resistance [81]. In contrast to serial tissue biopsies—which are invasive and often limited by tumor location and size—CTCs provide a minimally invasive, real‐time window into tumor dynamics [34]. Enriched with a spectrum of dynamic biomarkers, CTCs enable ongoing monitoring of treatment efficacy and allow for timely adaptation of therapeutic strategies [36]. In small cell lung cancer (SCLC), CTCs from both chemotherapy‐sensitive and chemotherapy‐resistant patients have demonstrated tumorigenic potential in immunodeficient mice. The resulting CTC‐derived xenografts recapitulated donor‐specific responses to platinum and etoposide treatment, underscoring the clinical relevance of CTC‐based models [44]. Molecular profiling of CTCs has also revealed treatment‐induced alterations that may guide therapeutic decisions [82]. For example, in patients with head and neck squamous cell carcinoma (HNSCC) treated with nivolumab, cyclin D1 (CCND1) expression was significantly downregulated, while NANOG expression was elevated—changes that may reflect therapeutic efficacy or resistance mechanisms [82]. Together, these findings highlight the critical role of CTC enumeration and molecular analysis in monitoring drug response and tailoring cancer treatment. The growing body of research on CTCs underscores their utility in companion diagnostics, prognostic assessment, and the real‐time evaluation of tumor heterogeneity, metastatic potential, and therapeutic outcomes.

Looking ahead, further investigation into the molecular mechanisms and clinical applications of CTCs will be essential for refining cancer diagnostics and treatment. As technologies advance, CTCs are poised to become an integral component of precision oncology—enhancing clinical decision‐making, improving patient quality of life, and extending survival.

## 4 Overview of CTC Enrichment Technologies Based on Micro/Nanomaterials and Microfluidic Diagnostic Platforms

The extreme rarity of CTCs in peripheral blood has long presented both a challenge and an opportunity for researchers, driving significant focus toward the development of efficient CTC enrichment technologies [83]. Due to the profound heterogeneity of tumors and the transitional epithelial–mesenchymal phenotypes exhibited by CTCs, identifying a universal biomarker for their capture remains elusive. Currently, CTC enrichment strategies fall into two main categories: immunoaffinity‐based approaches mediated by functionalized nanomaterials, and label‐free methods driven by microfluidic technologies (Figure 3). The former enhances target specificity through surface chemical modifications and topographical engineering, while dynamic regulation of ligand density and multivalent binding significantly improves capture efficiency across heterogeneous CTC subpopulations. The latter relies on optimized micro/nanostructures and fluid dynamics to exploit physical properties such as size, inertia, deformability, and electrical charge, enabling high‐efficiency, non‐destructive isolation while preserving cellular viability for downstream analyses. This section provides a comprehensive overview of recent advances in CTC enrichment technologies based on micro/nanomaterials and microfluidic diagnostic platforms, with a particular focus on the application and future potential of microfluidic membrane filtration platforms in CTC isolation.

**FIGURE 3:**
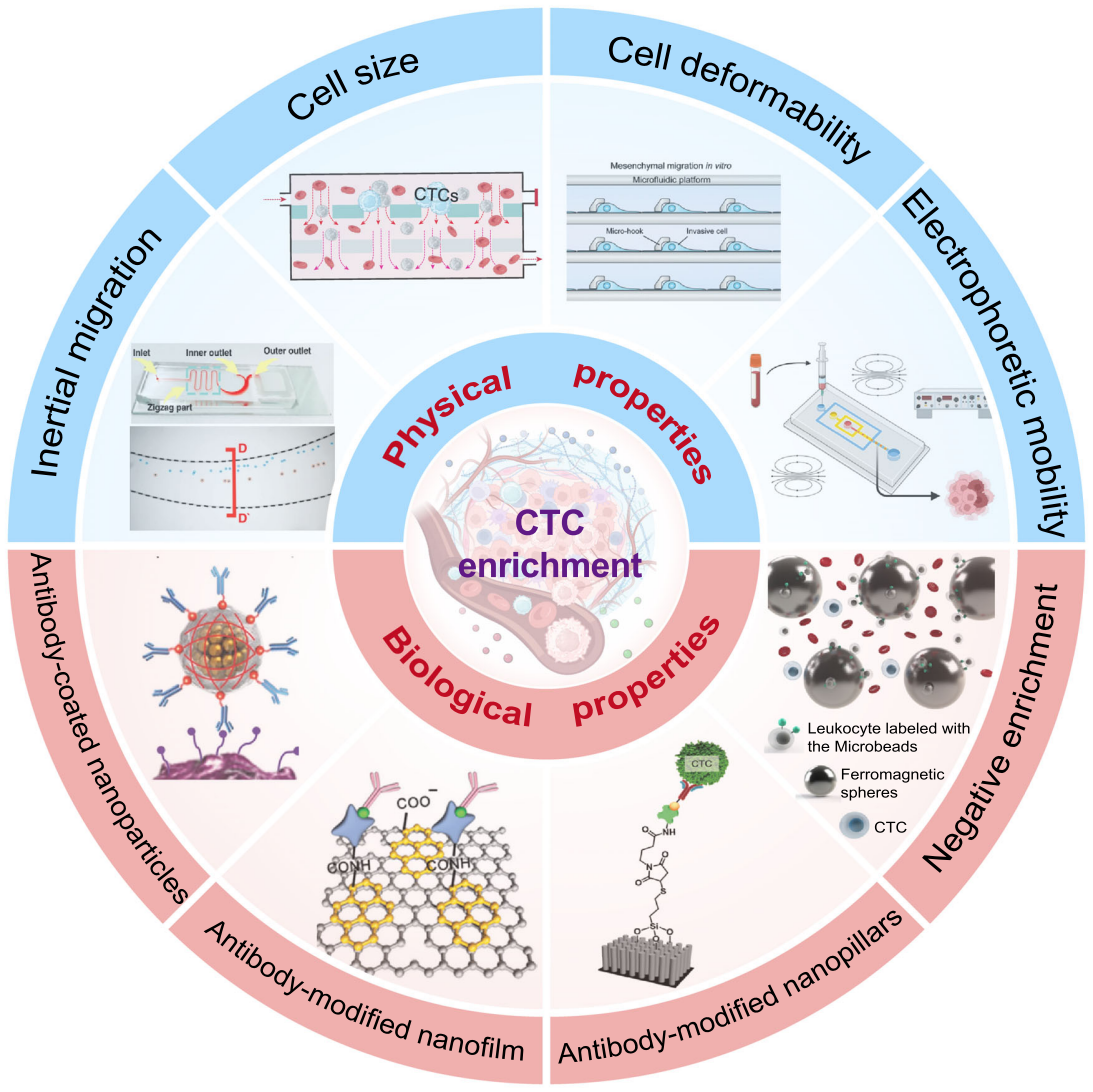
Summary of strategies for CTC enrichment. CTCs can be isolated using strategies based on physical or biological properties. Physical property‐based methods leverage differences in size, deformability, inertial migration, and electrical charge, often integrated with microfluidic platforms for enhanced separation efficiency. Biologically based enrichment typically involves immunoaffinity approaches using antibodies targeting epithelial surface markers such as EpCAM, which can be immobilized on functionalized nanoparticles, nanofilms, or nanostructured pillars and wires. Negative selection strategies remove leukocytes using anti‐CD45‐coated magnetic beads, while alternative techniques include rosette formation driven by erythrocyte‐leukocyte crosslinking.

### 4.1 Immunoaffinity Strategies Based on Biological Properties

Immunoaffinity strategies for CTC enrichment exploit tumor‐specific biological features to selectively isolate rare CTCs from a background of billions of blood cells [84]. Given that epithelial cells are exceedingly rare in healthy individuals but detectable in patients with malignancies, epithelial markers such as cytokeratins (CK18/19) and EpCAM are commonly targeted for CTC capture [85]. Figure 4 summarizes key advancements in immunoaffinity‐based technologies. These approaches include positive selection using antibodies or aptamers conjugated to magnetic beads, nanofilms, or micropillar arrays designed to capture CTCs with high specificity. Alternatively, negative selection strategies utilize materials functionalized with antibodies targeting leukocyte‐specific antigens (e.g., CD45) to deplete background cells while sparing CTCs [15, 16, 20, 86, 87]. Both strategies have demonstrated strong performance in enhancing the yield and purity of CTCs, offering valuable platforms for downstream analysis and clinical translation.

**FIGURE 4:**
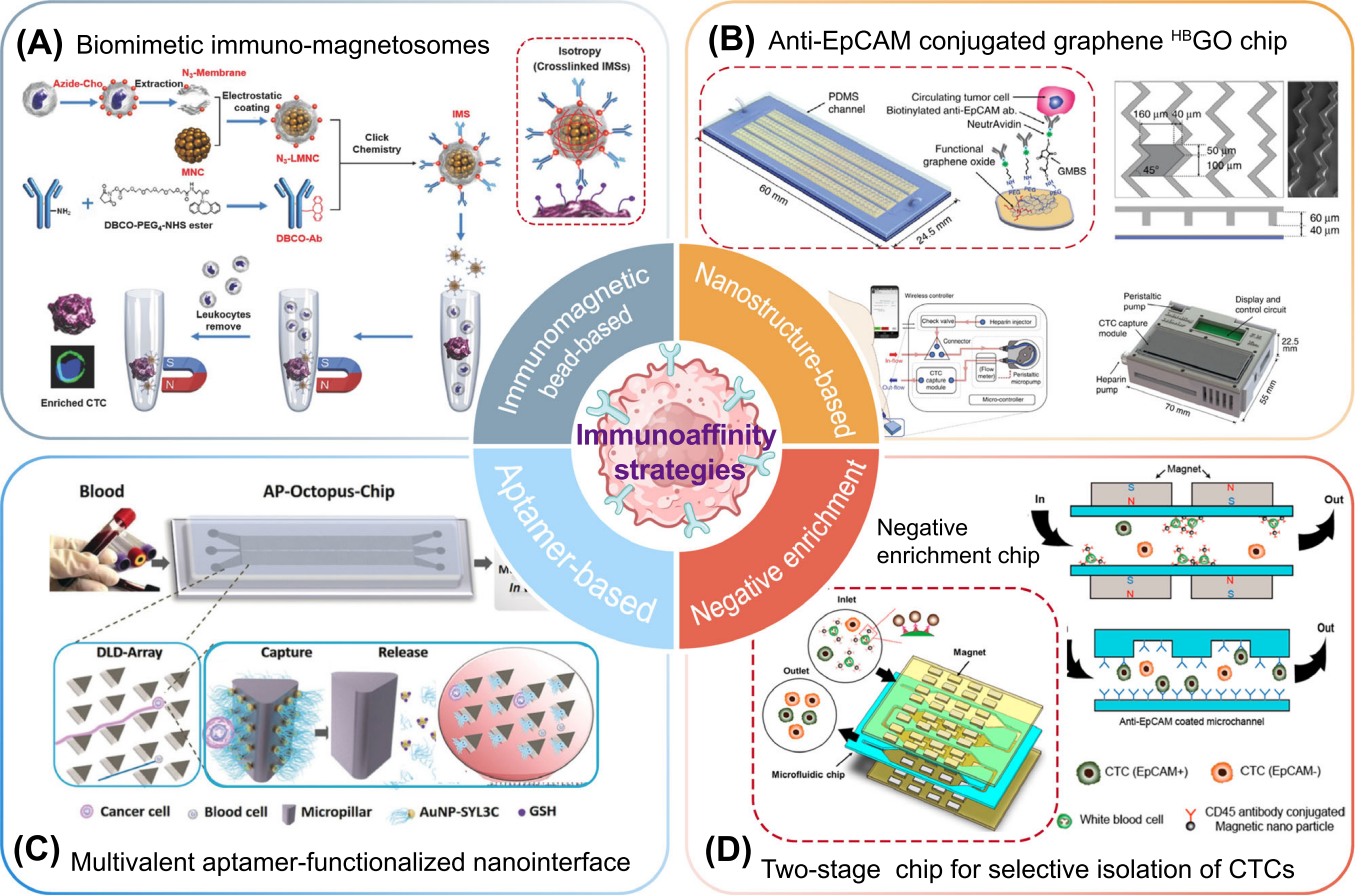
Advances in immunoaffinity‐based CTC enrichment strategies. (A) Biomimetic immunomagnetic spheres (IMS) enable high‐purity capture of CTCs through enhanced binding efficiency. Reproduced with permission [15]. Copyright 2016, Wiley‐VCH Verlag GmbH & Co. KGaA, Weinheim. (B) An intravascular in vivo CTC isolation device integrates EpCAM‐functionalized graphene oxide (GO) nanosheets for efficient and viable CTC enrichment directly from the bloodstream. Reproduced under the terms of the Creative Commons CC BY license [86]. Copyright 2019, The Author(s). (C) The AP‐Octopus‐Chip employs aptamer‐based tentacle‐like structures to achieve high‐efficiency capture and controlled release of CTCs. Reproduced with permission [20]. Copyright 2019, Wiley‐VCH Verlag GmbH & Co. KGaA, Weinheim. (D) A second‐generation microfluidic chip combines negative and positive enrichment strategies for selective and effective CTC isolation. Reproduced with permission [16]. Copyright 2014, Elsevier B.V.

Both manual and automated CTC enrichment systems based on marker expression have been described previously, with the CellSearch system being the first FDA‐approved platform for CTC detection [88]. This method uses EpCAM‐targeted antibodies conjugated to magnetic beads to isolate CTCs from whole blood. However, each CellSearch‐enriched sample typically retains 1,000‐3,000 leukocytes, resulting in a purity of less than 0.5%. This low specificity necessitates additional technologies to accurately identify rare CTCs amidst a high leukocyte background [89]. Moreover, suboptimal magnetic control and limited CTC recognition further compromise enrichment performance [15].

To address these limitations, Xiong et al. [15] developed a biomimetic immunomagnetic sphere (IMS) system, wherein leukocyte membranes were coated onto magnetic nanoclusters and further functionalized with EpCAM antibodies. This strategy conferred strong affinity for CTCs while simultaneously providing immunological camouflage from leukocytes, enabling efficient capture of rare CTCs in leukocyte‐dense samples (Figure 4A). Nevertheless, the presence of magnetic beads on CTC surfaces may interfere with downstream applications such as immunofluorescence imaging.

Alternatively, antibody‐functionalized nanomaterials offer an appealing solution. Their high surface area and rough texture promote strong contact with CTCs, enhancing capture affinity [87]. For instance, Kim et al. [86] developed an intravascular CTC isolation system that incorporated EpCAM‐functionalized graphene oxide (GO) nanosheets. This implantable device enabled highly efficient and viable CTC enrichment from large blood volumes over extended periods—surpassing the capacity of traditional blood draws (Figure 4B).

While antibodies have been widely used as CTC‐capture ligands, aptamers offer several advantages: smaller size, ease of modification, low cost, and high batch‐to‐batch consistency [90, 91]. Inspired by the multivalent tentacles of octopuses, Song et al. [20] designed the AP‐Octopus‐Chip using the EpCAM‐targeting aptamer SYL3C. Gold nanoparticles (AuNPs) served as scaffolds, forming a rough interface on which aptamers were immobilized. This topology enhanced local interactions and significantly improved capture efficiency (Figure 4C) [20].

Despite these advances, affinity‐based positive selection may miss subpopulations of CTCs undergoing EMT, such as EpCAM^low^ or EpCAM^−^ cells, thus limiting representation of tumor heterogeneity. To overcome this, negative enrichment strategies have been developed. As shown in Figure 4D, Hyun et al. [16] constructed a two‐stage microfluidic platform for high‐throughput, high‐purity CTC isolation. In the first stage, leukocytes were depleted using a negative selection chip; in the second, heterogeneous CTCs were captured and classified using a positive selection module.

In summary, affinity‐based CTC capture technologies offer high specificity and preserve cell viability but still face challenges related to capture efficiency, tumor heterogeneity, and cost. Continued refinement of these platforms will be critical for their broader clinical translation.

### 4.2 Label‐Free Strategies Based on Physical Properties

The dynamic phenotypic shifts that tumor cells undergo during EMT pose a major challenge to immunoaffinity‐based enrichment [92]. Specifically, EMT‐associated downregulation of epithelial markers such as EpCAM can significantly compromise the effectiveness of EpCAM‐targeted capture strategies [93]. Recognizing that these EMT‐like CTCs often possess high metastatic potential yet evade detection by conventional immunocapture, researchers have turned to label‐free enrichment methods that exploit inherent physical differences between CTCs and normal blood cells [94].

CTCs of epithelial origin typically differ from peripheral blood cells in several biophysical parameters—including size, density, electric charge, and deformability—providing a foundation for marker‐independent enrichment techniques. As shown in Figure 5, these label‐free approaches have evolved into diverse platforms, such as electrophoretic separation [95], inertial microfluidics [96, 97], acoustic‐based sorting [98, 99], deformability‐based filtration [100], and membrane‐based filtration of fixed samples [14]. Such methods offer the potential to capture a broader spectrum of CTC phenotypes, including those overlooked by immunoaffinity methods, thereby enabling a more comprehensive analysis of tumor heterogeneity.

**FIGURE 5:**
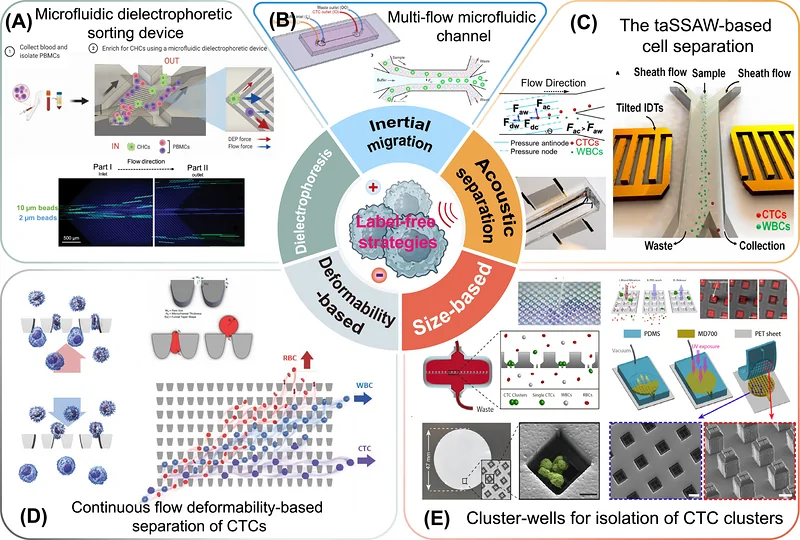
Advances in label‐free, physical property–based CTC enrichment strategies. (A) A label‐free dielectrophoretic microfluidic platform enables CTC isolation while maintaining compatibility with downstream phenotypic and genotypic analyses. Reproduced under the terms of the Creative Commons CC BY license [95]. Copyright 2021, The Author(s). (B) A novel high‐throughput inertial microfluidic system enriches CTCs with high purity across multiple flow streams. Reproduced under the terms of the Creative Commons CC BY license [97]. Copyright 2019, The Author(s). (C) An acoustic‐based CTC separation platform enables gentle and efficient sorting using sound waves. Reproduced with permission [99]. Copyright 2015, The Author(s) (D) A continuous‐flow microfluidic ratchet device leverages cell deformability to selectively isolate CTCs. Reproduced with permission [100]. Copyright 2016, Wiley‐VCH Verlag GmbH & Co. KGaA, Weinheim. (E) A high‐throughput size‐based mesh micropore system captures CTC clusters without labeling, enabling intact recovery and analysis. Reproduced under the terms of the Creative Commons CC BY license [14]. Copyright 2022, The Author(s).

The extreme rarity of CTCs presents a significant barrier to their use in multi‐omic analyses that reflect tumor heterogeneity [101]. To address this, Mira et al. [95] developed a label‐free dielectrophoresis (DEP) microfluidic platform that exploits the intrinsic electrical properties of cells for high‐throughput, rapid CTC enrichment, while maintaining compatibility with downstream phenotypic and genotypic analyses (Figure 5A). Moreover, cell size plays a crucial role in lateral migration within microchannels, enabling label‐free separation of CTCs from white blood cells (WBCs) without complex sample preparation or reliance on affinity‐based markers [96]. For example, Zhou et al. [97] introduced a multi‐stream inertial microfluidic device that achieves high‐purity (>87%) CTC enrichment without labeling (Figure 5B).

Among the emerging separation technologies, acoustofluidic methods offer compelling advantages due to their contact‐free, label‐free nature, preserving the phenotypic and genetic integrity of isolated cells [98, 99, 102]. Li et al. [99] reported an optimized acoustic separation platform that significantly outperformed previous tilted‐angle standing surface acoustic wave (taSSAW) systems, increasing CTC throughput by up to 20‐fold while achieving 83% separation efficiency (Figure 5C).

CTCs derived from cancers such as prostate and colorectal often display marked polymorphism and size overlap with leukocytes. However, their larger nuclei result in higher nuclear‐to‐cytoplasmic ratios and distinct deformability profiles [103, 104]. Park et al. [100] leveraged these differences to develop a label‐free method for deformability‐based enrichment of viable CTCs directly from whole blood (Figure 5D). This microfluidic system uses tapered micro‐constrictions and oscillatory flow to generate a ratchet effect that discriminates cells based on their deformability—even when their sizes are nearly identical [100].

Lastly, filtration‐based separation remains attractive due to its simplicity, cost‐effectiveness, and ease of integration. Boya et al. [14] introduced Cluster‐Wells, a platform combining the speed and practicality of membrane filtration with the precision of microfluidics (Figure 5E). Featuring over 100,000 microscale wells, Cluster‐Wells enables gentle, label‐free capture of intact CTC clusters from unprocessed whole blood at flow rates exceeding 25 mL h^−1^, while preserving cell viability and enabling cluster retrieval for downstream analysis.

Notably, the enrichment strategies discussed above can exhibit pronounced biases in capturing single CTCs versus CTC clusters, a factor that must be carefully considered in both device design and data interpretation. For example, size based filtration or microfluidic approaches (Section 4.2) tend to favor the capture of larger CTC clusters. However, their complex architectures also increase the risk of membrane clogging or excessive retention within micropillar arrays, which in turn compromises throughput and the stability of recovery rates. By contrast, EpCAM based immunoaffinity capture strategies (Section 4.1) rely on antigen accessibility at the cluster surface. Cells located within the interior of a cluster may be shielded by steric hindrance, reducing effective binding and leading to systematic underestimation.

Such detection biases carry direct clinical implications. CTC clusters are widely regarded as having higher metastatic potential and being associated with poorer prognosis. Platforms that systematically miss or underestimate clusters therefore risk failing to reflect the true disease burden and to accurately stratify patient risk. Future platform design should adopt more integrated strategies, such as combining size based selection with immunological recognition, together with cluster specific standards, to enable unbiased and efficient enrichment of both single CTCs and CTC clusters.

### 4.3 Membrane Filtration Technologies Based on Cell Size

Among the various physical‐property‐based methods [14, 95, 96] for CTC isolation—including those leveraging size, deformability, electrochemical features, dielectrophoresis, and inertial migration [98, 99, 104]—membrane filtration stands out for its simplicity, low cost, and ease of integration [105, 106, 107]. These systems typically consist of three‐dimensional housing structures incorporating membranes with precisely defined pore shapes and sizes, and can be readily combined with other enrichment strategies [108, 109]. Multiple studies have reported that size‐based isolation techniques, such as Isolation by Size of Epithelial Tumor cells (ISET), outperform the CellSearch system in clinical evaluations across several cancer types [110, 111, 112].

Despite their clinical feasibility, size‐based microfiltration approaches often struggle to balance capture efficiency, purity, and cell viability—key performance metrics for both research and diagnostic applications [113]. Major challenges include pore clogging and cell aggregation, which can compromise selectivity and impede downstream analyses such as fluorescence imaging.

As illustrated in Figure 6, the evolution of micro‐ and nanofabrication technologies has transformed membrane design from randomly distributed circular pores to uniform, structured pore arrays, enhancing CTC capture efficiency, purity, and recovery [17, 108, 109, 110, 113, 114]. Initially, polycarbonate (PC) membranes were widely used due to their low cost and user‐friendly format. In a direct comparison, Farace et al. [110] demonstrated that ISET outperformed CellSearch in detecting EpCAM^low^ CTCs, particularly in patients with metastatic lung cancer. However, the performance of PC membranes remains limited by their random pore distribution, susceptibility to clogging, and overlapping pore structures.

**FIGURE 6:**
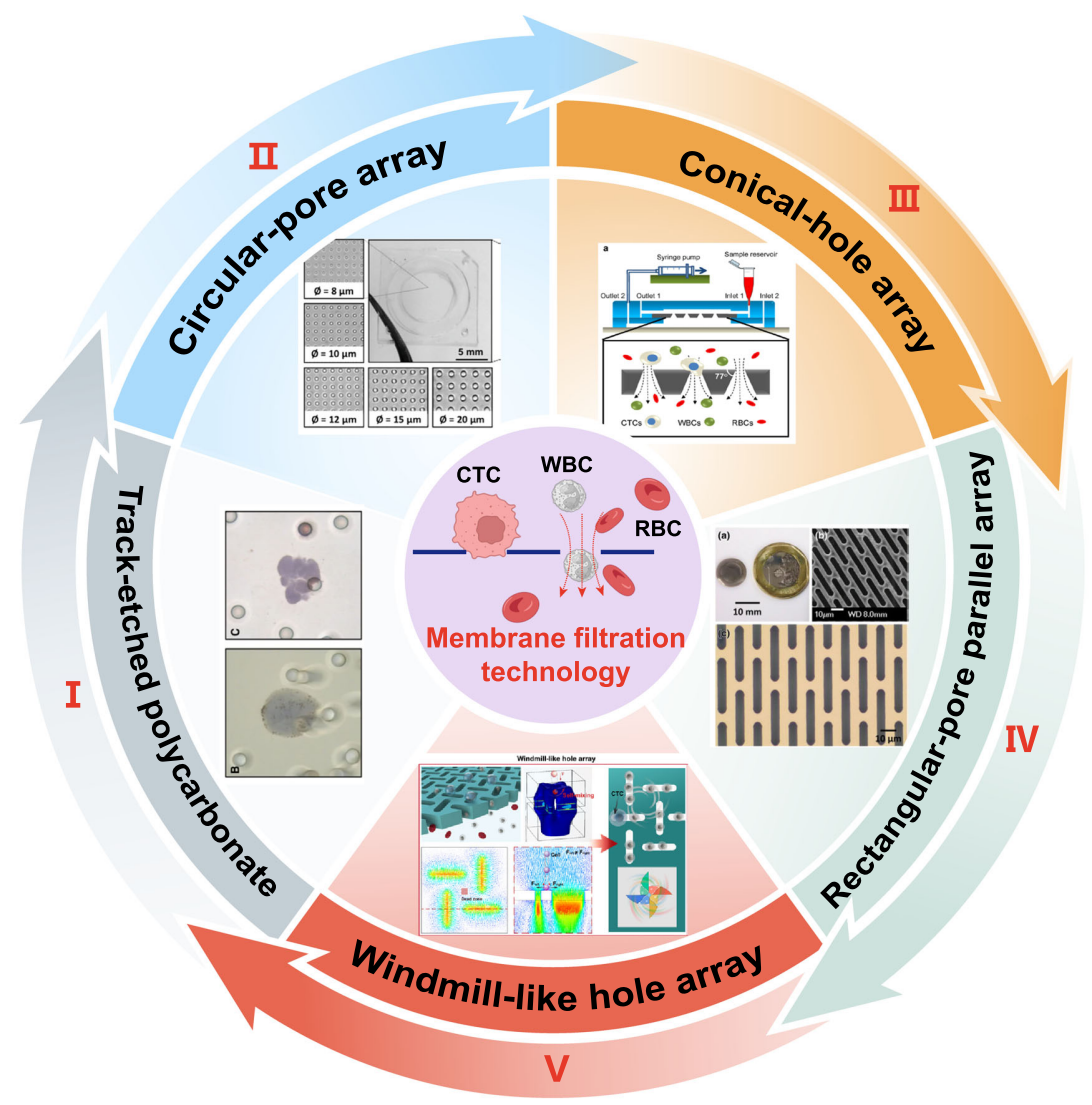
Advances in membrane filtration technologies for CTC enrichment. With the advancement of micro‐ and nanofabrication techniques, membrane pore architectures have evolved from disordered to highly ordered structures and from low to high porosity designs. The progression includes: **(I)** PC membranes with randomly distributed pores; reproduced under the terms of the Creative Commons CC‐BY‐NC‐SA license [110]. Copyright 2011, The Author(s). **(II)** cylindrical pore arrays; reproduced with permission [108]. Copyright 2016, American Chemical Society. **(III)** tapered pore arrays that enhance flow and reduce clogging; reproduced under the terms of the Creative Commons CC‐BY‐NC‐ND license [113]. Copyright 2014, The Author(s). **(IV)** rectangular pore arrays that improve leukocyte exclusion and preserve cell viability; reproduced with permission [109]. Copyright 2014, Springer Science Business Media New York. **(V)** windmill‐like pore arrays designed for high‐efficiency CTC capture with minimal mechanical stress. Reproduced under the terms of the Creative Commons CC BY license [17]. Copyright 2022, The Author(s).

Recent advances in microfabrication now allow the production of membranes with highly controlled pore size, shape, and density. For example, Meunier et al. [108] developed a 3D‐printed filtration cartridge containing microfabricated polymer membranes with cylindrical pores of 8, 10, 12, 15, or 20 µm. At a flow rate of 100 µL min^−1^, the system achieved >80% CTC recovery with low leukocyte contamination. Similarly, Tang et al. [113] engineered a microfluidic chip featuring tapered pore arrays. Compared to cylindrical pores, tapered geometries prevented clogging, minimized mechanical stress on cells, and delivered improved performance—achieving approximately 96% leukocyte depletion and 95% CTC viability. Further optimization was demonstrated by Hosokawa et al. [114], who replaced circular microcavity arrays with rectangular arrays, substantially reducing leukocyte carryover. The system's low‐pressure operation at reduced flow rates preserved cell viability, illustrating how refined geometry and flow control can improve both performance and biological integrity in size‐based CTC isolation platforms.

From the early use of PC membranes with uneven pore distribution to the later adoption of cylindrical, tapered, and rectangular pore arrays, advances in membrane microarchitecture have steadily improved leukocyte depletion efficiency in CTC enrichment. However, pore clogging and cell aggregation remain persistent challenges, significantly limiting the capture efficiency and detection accuracy of CTCs. Table 1 summarizes the comparative performance of size‐based membrane filtration technologies developed in recent years. To mitigate clogging, blood samples are typically diluted prior to filtration. Flow rate is another critical parameter, directly influencing capture efficiency and the uniform dispersion of cells across the membrane. As cells accumulate on the membrane surface, higher flow rates inevitably increase transmembrane pressure, raising the risk of mechanical damage and cellular rupture during passage through the pores. In general, lower flow rates and higher dilution volumes favor optimal CTC recovery and cell integrity—as reflected in Table 1—but these conditions often conflict with the need for rapid processing in clinical settings. Addressing this bottleneck, Li et al. [17] introduced a biomimetic membrane inspired by windmill perturbation patterns observed in nature. The design incorporates windmill‐like hole array that induce self‐disturbance and fluid mixing during flow, enabling uniform, high‐throughput, and clog‐free CTC enrichment. Moreover, the orderly arrangement of cells on the membrane surface facilitates downstream applications such as immunofluorescence imaging and molecular profiling, positioning this platform as a promising tool for both clinical and research applications.

**TABLE 1:** Comparative performance of recent membrane filtration technologies.

Device	Membrane type	Pore size & arrangement	CTC recovery	CTC purity	Flow rate
ScreenCell [110]	Track‐etched PC membrane	7.5 µm circular pores, randomly distributed	74% (2 spiked); 91.2% (5 spiked)	∖	1.2 mL min^−1^
3D‐printed filter box [108]	Transparent polymer membrane	8 µm circular pore array	>80%	< 1000 WBCs contamination	0.1 mL min^−1^
Microfluidic chip with tapered flow control [113]	PEGDA	5.5/6.5/8.0 µm tapered pore arrays	∼95%	∼96% WBC removal	0.2 mL min^−1^
Integrated PMM microdevice [115]	PMM	9.1 µm circular pore array	>90%	∖	10 mL h^−1^
MCA [114]	Electroformed metal membrane	8×30 µm rectangular pores, aligned array	∼80%	854 ± 306 WBCs /mL	0.2 mL min^−1^
Microfluidic platform for negative enrichment [109]	Parylene C	5.5×40 µm microcracks, aligned array	>90%	2.3 log_10_ WBC depletion	∼0.8 mL min^−1^
Integrated microdevice with windmill‐like hole array [17]	SU‐8	7×35 µm windmill‐like hole array	93% (A549 cells);90% (HeLa cells)	98.7% WBC removal	2 mL min^−1^

In summary, the choice of CTC enrichment strategy not only determines capture efficiency and purity but also directly shapes the feasibility, quality, and dimensionality of downstream multi‐omics analyses. Immunoaffinity‐based methods, which rely on specific surface markers such as EpCAM, offer high specificity but introduce inherent analytical bias—potentially missing CTC subpopulations that have undergone EMT, thereby underrepresenting tumor heterogeneity. Moreover, surface‐immobilized antibodies or enzymatic release steps may interfere with proteomic analyses or compromise cell viability, limiting functional studies.

By contrast, label‐free enrichment strategies based on physical properties—particularly membrane filtration—capture phenotypically diverse CTCs without marker dependence. This approach better preserves native cell states and membrane protein integrity, providing superior compatibility for downstream applications such as single‐cell sequencing, culture, and multimodal molecular profiling across DNA, RNA, and protein layers. Accordingly, CTC research workflows should be designed in reverse—from the analytical endpoint backward—to ensure that the chosen enrichment strategy best aligns with the intended downstream objectives.

## 5 Overview of CTC Release and Identification Technologies Mediated by Micro/Nanostructured Materials and Microfluidic Diagnostic Platforms

Once an enrichment strategy compatible with downstream analyses has been established, the next critical step lies in efficiently and non‐destructively releasing the captured CTCs—an essential prerequisite for their molecular and functional characterization. The choice of release strategy is largely dictated by the initial capture mechanism: immunoaffinity‐based approaches require disruption of antigen–antibody interactions, whereas label‐free physical methods rely on weakening physical adhesion or spatial confinement. Consequently, all release techniques discussed in this section are developed around a central objective—to achieve high recovery efficiency while preserving cell viability and molecular integrity, thereby ensuring high‐quality samples for the multidimensional characterization methods detailed in Section 5.2 and beyond.

### 5.1 Strategies for Releasing CTCs From Micro/Nanostructured Substrates

Micro/nanostructured substrates have demonstrated considerable advantages in the efficient capture of CTCs. However, the increasing demand for downstream molecular analysis and functional assays has underscored a central challenge in the field: how to recover captured cells while preserving their viability and minimizing contamination by background hematologic cells. This requires overcoming the adhesive forces employed during capture, including both affinity interactions between ligands and cell surface markers, and cell–substrate focal adhesion [116].

Notably, studies have shown that applying fluid shear stress up to 180 dyn/cm^2^ within microfluidic devices releases only ∼50% of antibody‐bound CTCs, often at the cost of substantial cellular damage [117]. Given the enhanced adhesion properties of nanostructured surfaces, similar approaches may require even greater forces, underscoring the urgent need for non‐destructive cell retrieval methods. Moreover, the prevalence of non‐specific leukocyte adhesion in integrated microfluidic–nanomaterial systems necessitates the development of selective CTC release strategies to streamline downstream workflows.

Current release techniques (Figure 7) can be broadly categorized into three groups: (i) laser microdissection [118]; (ii) stimuli‐responsive release (e.g., light‐ [119], heat‐ [120], electric‐ [121], or magnetically [122] triggered mechanisms); and (iii) reagent‐induced detachment (e.g., chemical reactions [123], enzymatic digestion [124], or DNA hybridization [125]). In some cases, spatially selective release can be achieved under fluorescence microscopy guidance. Key performance metrics include release efficiency (i.e., the ratio of recovered to captured cells), operational duration, and the viability and purity of retrieved CTCs. To minimize procedural complexity and cell damage, in situ strategies are also emerging as promising alternatives—directly lysing captured CTCs on‐chip for nucleic acid or protein extraction, or integrating biosensing elements such as microelectrode arrays or in situ PCR for real‐time molecular interrogation [17, 56, 126].

**FIGURE 7:**
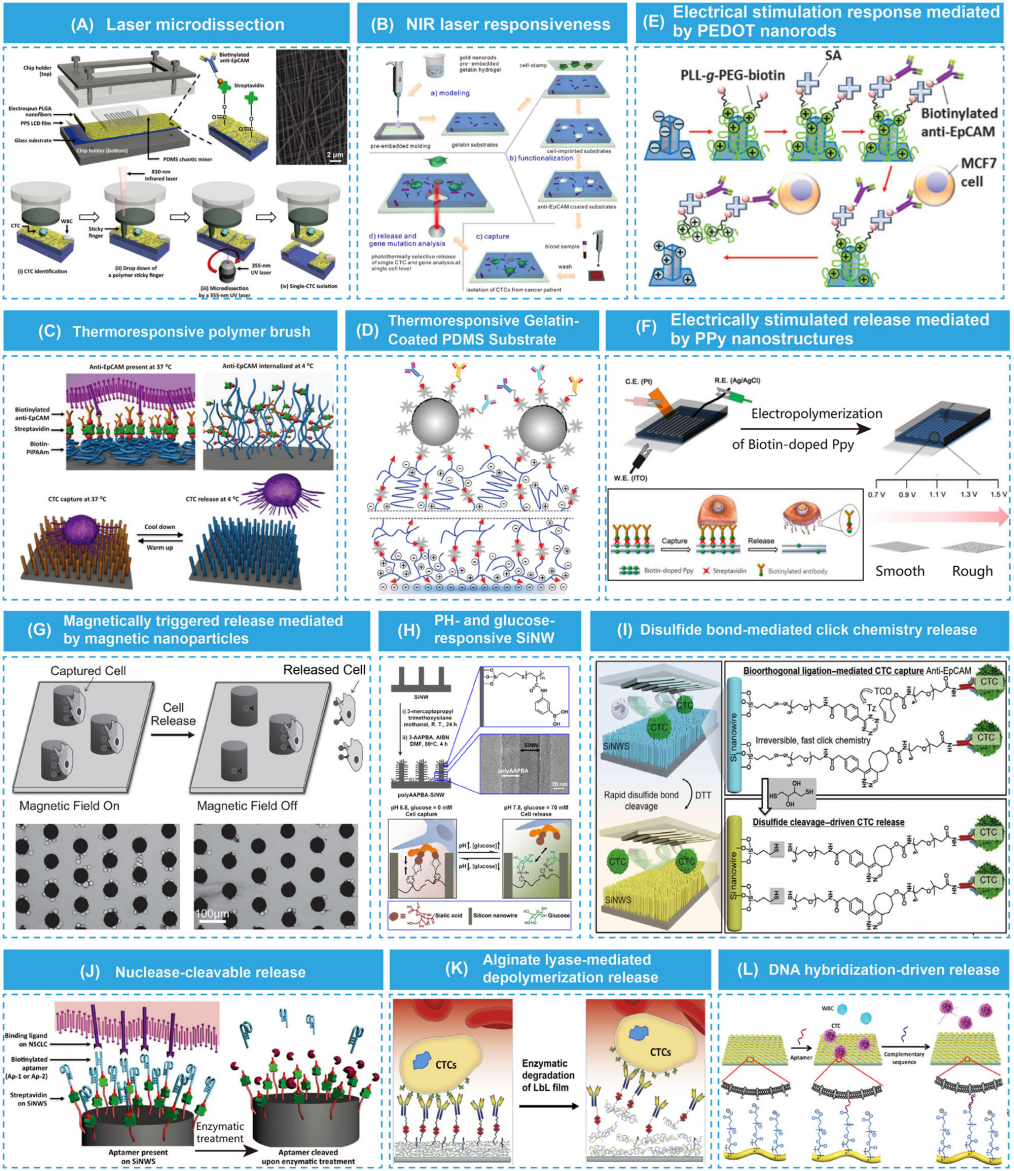
Various strategies for releasing CTCs from micro/nanostructured substrates. (A) Laser capture microdissection (LCM) from PN‐NanoVelcro substrates enable spatially selective recovery of individual CTCs, as shown in schematic and microscopy images (PN‐NanoVelcro/LCM). Reproduced with permission [127]. Copyright 2013, Wiley‐VCH Verlag GmbH & Co. KGaA, Weinheim. (B) Near‐infrared (NIR) responsive release: CTCs are captured and locally released via gold nanorods (GNRs) embedded within a gelatin substrate. Reproduced with permission [128]. Copyright 2016, American Chemical Society. (C) Thermo‐responsive NanoVelcro substrate: temperature‐mediated capture and release of CTCs are achieved through the exposure/internalization of EpCAM antibodies on polymer brushes‐binding at 37°C and release at 4°C. Reproduced with permission [120]. Copyright 2013, Wiley‐VCH Verlag GmbH & Co. KGaA, Weinheim. (D) Gelatin‐coated PDMS substrate enabling thermally controlled capture and release of CTCs. Reproduced with permission [129]. Copyright 2015, Wiley‐VCH Verlag GmbH & Co. KGaA, Weinheim. (E) Electrically triggered desorption‐based release of CTCs from a bioelectronic PEDOT nanorod substrate via PLL‐g‐PEG‐biotin linkage. Reproduced with permission [130]. Copyright 2015, Royal Society of Chemistry. (F) Electroresponsive capture and release using biotin–polypyrrole (Ppy) nanostructured substrates. Reproduced under the terms of the Creative Commons Attribution‐NonCommercial 4.0 International License (CC BY‐NC 4.0) [131]. Copyright 2014, Ivyspring International Publisher. (G) Magnetic field‐mediated capture and release of CTCs using GO‐Fe_3_O_4_ nanoparticle‐modified micropillar arrays. Reproduced with permission [122]. Copyright 2013, Wiley‐VCH Verlag GmbH & Co. KGaA, Weinheim. (H) pH‐ and glucose‐dual‐responsive SiNW‐embedded substrates for CTC capture and controlled release. Reproduced with permission [123]. Copyright 2013, American Chemical Society. (I) “Click Chip”: bioorthogonal chemistry enables CTC capture via click‐linkers and release via disulfide bond cleavage within a SiNW‐integrated microfluidic platform. Reproduced under the terms of the Creative Commons Attribution‐NonCommercial 4.0 International License (CC BY‐NC 4.0) [132]. Copyright 2019 The Authors, some rights reserved; exclusive licensee American Association for the Advancement of Science. (J) Enzymatic DNA aptamer digestion on NanoVelcro substrates enables selective release of NSCLC CTCs. Reproduced with permission [124]. Copyright 2013, Wiley‐VCH Verlag GmbH & Co. KGaA, Weinheim. (K) CTC capture and release on enzyme‐degradable alginate/poly (allylamine hydrochloride) (ALG/PAH) nanofilm substrates. Reproduced with permission [133]. Copyright 2015, Elsevier Ltd. (L) Aptamer‐based recognition and DNA hybridization‐triggered release on pCBMA–chitosan nanofiber substrates. Reproduced with permission [125]. Copyright 2016, Wiley‐VCH Verlag GmbH & Co. KGaA, Weinheim.

#### 5.1.1 Laser Microdissection

Laser microdissection (LMD) is a high‐precision, laser‐based physical separation technique well‐suited for isolating single cells adhered to transparent nanostructured substrates [118]. The core principle involves using a focused laser beam to cut the interface between the substrate and the target cell, enabling localized detachment with minimal disruption [127].

For instance, in the PN‐NanoVelcro platform (Figure 7A), researchers electro‐deposited poly (lactic‐co‐glycolic acid) (PLGA) nanofibers onto a substrate and functionalized them with anti‐CD146 antibodies to selectively capture circulating melanoma cells (CMCs) from patient blood samples [127]. A 355 nm ultraviolet (UV) laser was then employed to precisely ablate both the PLGA nanofibers and the underlying polyphenylene sulfide (PPS) membrane, releasing the captured cells. To improve operational efficiency, an infrared laser‐based capture microdissection (LCM) was developed using a thermally responsive “adhesive finger” approach (Figure 7A). By locally heating the substrate, this technique reduced electrostatic interference, significantly minimizing cell loss during CTC isolation in prostate cancer patients. Isolated single cells could be directly subjected to downstream molecular analyses such as Sanger sequencing.

Despite its precision, LMD/LCM technologies remain costly and technically demanding, with lengthy protocols that can compromise cell viability. Prolonged staining procedures and exposure to UV irradiation pose additional risks, potentially damaging cellular DNA and denaturing proteins, thus limiting the fidelity of downstream molecular profiling.

#### 5.1.2 Stimuli‐Responsive Release

Stimuli‐responsive strategies enable the controlled, gentle release of CTCs by dynamically modulating substrate surface properties in response to external physical cues—such as light, heat, electric fields, or magnetic forces. These approaches offer high spatiotemporal resolution and reversibility, allowing precise cell manipulation without compromising viability.

Near‐infrared (NIR) light‐triggered release leverages photothermal effects to locally liberate captured cells. As shown in Figure 7B, gelatin hydrogels embedded with gold nanorods (GNRs) and functionalized with anti‐EpCAM antibodies achieved efficient CTC capture (∼92%) [128]. Upon NIR laser irradiation, the GNRs convert light to heat, raising the local temperature to the gel–sol transition threshold of gelatin (∼32°C). The resulting solubilization of the gelatin matrix disassembles the capture interface, releasing CTCs from the targeted region with high efficiency (92%) and cell viability (90%). This technique offers exceptional spatial control with minimal cellular damage, making it ideal for retrieving rare cells from complex biological samples.

Thermoresponsive polymers provide a reversible mechanism for CTC capture and release via temperature‐dependent conformational changes. For example, Figure 7C illustrates a silicon nanowire (SiNW) substrate grafted with poly(N‐isopropylacrylamide) (PNIPAAm) brushes [120]. At 37°C, the polymer adopts a hydrophobic state that enhances anti‐EpCAM–CTC interactions. Cooling to 4°C induces a hydrophilic conformation, increasing steric hindrance around the antibodies and enabling gentle release of CTCs with a viability of up to 90%. Similarly, in a bulk‐release strategy (Figure 7D), gelatin coatings undergo sol‐gel transition at 37°C, disrupting cell–substrate adhesion and achieving release efficiencies up to 93% [129].

Electrochemical modulation offers rapid, on‐demand cell release by altering substrate surface chemistry. In one design (Figure 7E), a PEDOT (poly(3,4‐ethylenedioxythiophene)) nanorod substrate is functionalized with poly‐L‐lysine‐graft‐PEG‐biotin (PLL‐g‐PEG‐biotin) for antibody immobilization [130]. Application of cyclic voltages from –0.8 V to 0.5 V modifies the surface charge distribution, weakening biotin–streptavidin interactions and releasing CTCs with 87% efficiency while maintaining membrane integrity. Another platform using polypyrrole (Ppy) nanostructures (Figure 7F) achieves >90% release efficiency within 2–15 s via voltage‐induced contraction of the conductive polymer, which disrupts biotin–antibody linkages [131]. This approach allows precise control over release kinetics and supports high‐throughput recovery for downstream analysis.

Magnetic nanoparticles enable contactless manipulation of CTCs. As depicted in Figure 7G, a micropillar substrate modified with graphene oxide‐Fe_3_O_4_ (GO‐Fe_3_O_4_) composites facilitates CTC capture when an external magnetic field attracts anti‐EpCAM–conjugated magnetic beads to the surface, achieving >70% efficiency [122]. Upon removal of the magnetic field, the beads detach, releasing the captured cells. This simple and reversible method shows promise, though further optimization of nanoparticle biocompatibility is necessary to mitigate potential long‐term toxicity.

#### 5.1.3 Reagent‐Mediated Release

Reagent‐mediated strategies exploit molecularly specific interactions—such as chemical competition, enzymatic cleavage, or DNA hybridization—to disrupt the capture interface and achieve efficient CTC release. Their principal advantage lies in high selectivity, which substantially enhances release purity.

A representative example of chemical displacement involves competitive binding between boronic acids and saccharides. As illustrated in Figure 7H, silicon nanowires functionalized with poly(3‐acrylamidophenylboronic acid) (polyAAPBA) capture CTCs via boronic acid–sialic acid interactions under mildly acidic conditions (pH 6.8) [123]. Upon increasing the pH to 7.8, excess glucose competes with sialic acid for boronic acid binding sites, releasing CTCs with 95% efficiency and 96% viability. In another example, a “Click Chip” platform (Figure 7I) employs disulfide bond reduction to trigger release [132]. Treatment with 50 mM dithiothreitol (DTT) for 12 min cleaves disulfide linkages on the substrate, achieving 80% release efficiency and reducing leukocyte contamination from 2,000 to 200 cells—thus enabling high‐purity samples suitable for single‐cell mRNA profiling.

Enzyme‐mediated approaches rely on site‐specific degradation of biomolecular linkers within the capture interface. For instance, the NanoVelcro chip (Figure 7J) uses benzonase to digest DNA aptamers, releasing non‐small cell lung cancer CTCs with an efficiency of 85% and cell viability between 78% and 83% [124]. Similarly, in Figure 7K, alginate/poly(allylamine hydrochloride) (ALG/PAH) nanofilms undergo enzymatic degradation by alginate lyase, resulting in 95% release efficiency and 90% viability [133]. These systems require precise tuning of enzyme concentration and incubation time to avoid over‐digestion and potential cell damage.

DNA hybridization offers a programmable route to CTC release by inducing conformational changes in aptamers or displacing them via complementary oligonucleotides. As shown in Figure 7L, chitosan nanofiber substrates use DNA aptamers to capture CTCs, which are subsequently released through the introduction of complementary strands that disrupt aptamer–cell binding by unfolding the aptamer structure [125]. This method achieves a release efficiency of up to 98%. While highly specific, its success depends on the careful design of high‐affinity aptamers to minimize nonspecific adsorption.

The exceptional performance of micro‐ and nanomaterials in CTC capture and release stems from their finely tuned structure–function relationships. Physical topography—such as the vertical alignment of nanowires, the taper and arrangement of micropores, and surface roughness—directly modulates cell–substrate contact area and interfacial forces, thereby determining capture selectivity and efficiency. Meanwhile, surface chemical modifications, including the density, spatial orientation, and conjugation chemistry of antibodies or nucleic acid aptamers, govern molecular recognition specificity and binding affinity.

Yet, the pursuit of higher performance must be balanced against downstream analytical compatibility and biological safety. Residual magnetic nanoparticles or crosslinking agents, for instance, can strongly inhibit enzyme‐driven reactions such as PCR or sequencing. Likewise, non‐degradable nanostructures (e.g., silicon nanowires) or harsh lysis conditions—while not inherently toxic—can severely compromise cell viability, precluding single‐cell culture, drug sensitivity testing, and other functional assays.

Future development of functional materials should therefore prioritize three directions. First, design stimuli‐responsive materials that enable efficient, non‐destructive cell release under gentle external triggers such as near‐infrared light or specific biomolecular cues. Second, engineer bioinspired interfaces that enhance biocompatibility and minimize perturbation of the native cellular state. Third, construct integrated multifunctional platforms that seamlessly couple capture, release, and downstream molecular analysis. Such innovations will not only advance the standardization and clinical translation of CTC technologies but also help realize a new generation of intelligent, adaptive analytical systems for precision oncology.

### 5.2 Multidimensional Analytical Techniques for Released CTCs

As a dynamic and heterogeneous cell population, CTCs provide critical multidimensional insights into tumor biology across DNA, RNA, and protein levels. With advancing technologies, CTC analysis has evolved far beyond simple enumeration. Comprehensive molecular and functional profiling now enables detailed genotypic, transcriptomic, and phenotypic characterization of CTCs at both bulk and single‐cell resolution. The molecular landscape of CTCs can be interrogated using a suite of powerful techniques, including fluorescence in situ hybridization (FISH) and single‐cell sequencing for genomic alterations, reverse transcription PCR (RT‐PCR) and single‐cell RNA sequencing (scRNA‐seq) for transcriptomic dynamics, as well as immunofluorescence staining and mass spectrometry for proteomic and phenotypic profiling. These tools allow in‐depth interrogation of the molecular landscape of tumors, facilitating a better understanding of cancer biology and enhancing the diagnostic and therapeutic potential of liquid biopsy. This section reviews key technologies used for CTC identification at the genomic, transcriptomic, and proteomic levels. It further introduces emerging multidimensional analysis strategies and highlights the growing role of PCR‐based approaches in the integrated molecular profiling of CTCs.

#### 5.2.1 Genotypic Profiling of CTCs

Genomic instability is a hallmark of cancer evolution and underlies the emergence of distinct tumor subtypes [134]. In the context of metastasis and therapeutic resistance, monitoring such genomic alterations in real time is vital for precision oncology. By tracking dynamic genotypic changes in CTCs, clinicians can make more informed decisions, improving treatment outcomes and patient prognosis.

Mutation profiling of key oncogenes in CTCs is a central aspect of genotypic analysis. For instance, determining *EGFR* and *KRAS* mutation status is crucial for selecting EGFR tyrosine kinase inhibitors in non‐small cell lung cancer (NSCLC) and anti‐EGFR therapies in colorectal cancer. Digital PCR has proven effective in detecting these mutations in CTCs from NSCLC patients, showcasing its utility in monitoring therapeutic response through liquid biopsy [135, 136]. Importantly, genotyping CTCs requires highly pure and viable cells. Technologies such as the AP‐Octopus‐Chip developed by Song et al. enable gentle and efficient release of CTCs using aptamer‐functionalized gold nanoparticles, thereby preserving cell integrity for downstream analysis [20]. Using Sanger sequencing, *KRAS* mutations were identified in blood‐derived CTCs from colorectal and prostate cancer patients [20]. High‐throughput next‐generation sequencing (NGS) further enhances the depth of CTC genotyping. For example, Luca et al. used single‐cell NGS to analyze 25 genes in individual CTCs, identifying 51 sequence variants and revealing both inter‐ and intra‐patient heterogeneity. Somatic mutations in *TP53*—a gene frequently associated with poor prognosis in breast cancer—were particularly prevalent [137].

Copy number variation (CNV) analysis in CTCs offers additional insight into tumor evolution [138]. Although NGS‐based CNV profiling requires extensive DNA amplification from minute samples, conventional exponential amplification techniques such as PCR or multiple displacement amplification (MDA) can introduce bias [139]. To address this, Kim et al. developed a rapid emulsified droplet‐based MDA (re‐ddMDA) method, achieving uniform amplification suitable for high‐fidelity CNV detection in single CTCs [140].

Other genotyping methods include FISH, which enables detection of drug‐sensitivity markers in CTCs, such as *ALK* rearrangements [141] in NSCLC and *HER2* amplification [142] in breast cancer. Aneuploidy, another feature of malignancy, can also be evaluated in CTCs. Wang et al. introduced a subtraction enrichment–integrated immunostaining FISH (SE‐iFISH) platform that combines phenotypic and karyotypic analysis of HCC CTCs [143]. SE‐iFISH allows subtyping of CTCs based on both tumor marker expression and chromosomal ploidy—subtypes that may correlate with distinct clinical outcomes [143].

However, discordance between CTCs and matched primary tumors remains a key challenge. For example, in colorectal cancer, the concordance rate of *KRAS* mutations between CTCs and primary tumors has ranged from 37% to 90% [144]. Similar discrepancies have been observed in prostate [145], breast [146], liver cancers [10] and so on. Even in breast cancer, CNV profiles often differ between CTCs and matched metastatic lesions [147]. These inconsistencies likely stem from tumor heterogeneity as well as variable detection efficiencies across analytical platforms. To overcome these limitations, integrating genotypic data with transcriptomic and phenotypic analyses offers a more comprehensive and accurate view of the tumor landscape. Multidimensional profiling enhances the precision of cancer diagnostics and enables the tailoring of more effective, individualized treatment strategies.

#### 5.2.2 Transcriptomic Profiling of CTCs

Transcriptomic analysis of CTCs provides critical insight into gene expression dynamics, revealing the regulatory landscape underlying cellular processes, development, and responses to external stimuli. Advances in high‐throughput transcriptomics have enabled large‐scale expression profiling, and among the most powerful tools is scRNA‐seq, which allows for the interrogation of rare cell populations and the dissection of intratumoral heterogeneity.

scRNA‐seq workflows typically begin with the isolation of individual cells using robotic micromanipulation, fluorescence‐activated cell sorting (FACS), or microfluidic platforms. To mitigate amplification bias, unique molecular identifiers (UMIs) are added to RNA molecules prior to linear cDNA amplification via in vitro transcription. The resulting cDNA libraries are barcoded and sequenced using NGS.

For example, Sun et al. [148] performed full‐length single‐cell RNA sequencing on 113 CTCs isolated from four vascular compartments—hepatic vein, peripheral artery, peripheral vein, and portal vein—and identified CCL5 as a key mediator of immune evasion. This study uncovered previously unrecognized spatial heterogeneity and immune escape mechanisms of CTCs. However, the technical complexity and high cost of sequencing pose challenges for routine longitudinal monitoring.

To address this, several groups have developed RT‐PCR assays targeting specific CTC‐associated transcripts [149, 150]. In one study, Sun et al. combined CellSearch‐based four‐channel immunofluorescence with microfluidic RT‐qPCR to analyze EMT markers in CTCs across different vascular sites, revealing pronounced spatial heterogeneity in CTC distribution and phenotype [150]. In another approach, Kalinich et al. employed whole transcriptome amplification (WTA) of CTC‐derived RNA in conjunction with droplet digital PCR (dPCR). In this setup, cDNA molecules were encapsulated in individual droplets, and multiple transcripts of interest were quantified simultaneously, enabling highly sensitive and lineage‐informed cancer detection from blood samples [149].

MicroRNAs (miRNAs), potent regulators of gene expression, have also emerged as promising diagnostic markers and therapeutic targets [151]. Gasch and colleagues developed a robust in situ hybridization (ISH) protocol integrated with the CellSearch system, enabling single‐cell‐level miRNA analysis in CTCs. In a proof‐of‐concept study, they demonstrated heterogeneous expression of miR‐10b in individual CTCs from patients with breast, prostate, and colorectal cancers [152].

While transcriptomic profiling offers valuable insights into tumor heterogeneity and cell‐state dynamics, RNA‐based signatures alone reflect only potential biological activity and may not fully capture the functional consequences of molecular alterations. Future research should prioritize integrated, multi‐omic strategies that combine genotypic, transcriptomic, and phenotypic data to construct a more complete molecular portrait of CTCs and their role in tumor evolution.

#### 5.2.3 Phenotypic Profiling of CTCs

Proteins, as the primary executors of cellular function and structure, are central to understanding tumor behavior and therapeutic response. Phenotypic profiling of CTCs—through surface, intracellular, and secreted protein expression—offers crucial insights into tumor progression, metastasis, and treatment efficacy.

Immunofluorescence staining and enumeration remain widely used techniques for characterizing CTC phenotypes [14, 89, 94, 99]. Standard markers include positive staining for epithelial adhesion molecules such as EpCAM or cytokeratins (CK18/19), and negative staining for leukocyte marker CD45. Most protocols define CTCs as EpCAM^+^/CD45^−^/DAPI^+^ cells, using these criteria to evaluate enrichment performance or disease burden. However, during EMT and MET, CTCs often downregulate epithelial markers, making them difficult to detect by conventional immunostaining. Furthermore, fluorescence‐based methods are limited in sensitivity and multiplexing capability, and CTC enumeration alone provides only a coarse assessment of protein expression.

Beyond surface and secreted proteins, profiling intracellular targets is equally critical, particularly for dissecting metastatic behavior and tumor heterogeneity. Abdulla et al. developed an integrated microfluidic platform enabling label‐free CTC isolation and single‐cell immunoblotting (ieSCI) [96]. This platform integrates single‐cell Western blotting (scWB), combining microfluidic precision with the resolving power of SDS‐PAGE to achieve high specificity and multiplex detection—circumventing spectral overlap and antibody cross‐reactivity that limit immunofluorescence [96].

In another study, Sinkala et al. applied an eight‐protein scWB panel to analyze single CTCs from estrogen receptor–positive (ER^+^) breast cancer patients [153]. This method allowed relative quantification of both surface and intracellular signaling proteins in individual CTCs, enabling assessment of phenotypic heterogeneity across cells. Such proteomic profiling offers a complementary classification framework, advancing our understanding of CTC biology and potential clinical implications [153]. Nonetheless, scWB is largely limited to qualitative or semi‐quantitative analysis, and its sensitivity remains a technical hurdle.

Quantitative immuno‐PCR (qIPCR) offers a promising solution by combining the specificity of immunoassays with the exponential amplification capacity of PCR. Mei et al. demonstrated a qIPCR method using DNA‐tagged anti‐CK19 antibodies to quantify CTCs enriched via EpCAM‐magnetic bead separation, achieving a detection rate of 92.3% in clinical tumor blood samples [154]. By quantifying the tagged DNA anchored to tumor cells, the authors demonstrated a clear correlation between CTC abundance and disease stage or progression. Building on this concept, GenoSaber Biotech developed the ligand‐targeted PCR (LT‐PCR) platform, which directly links small‐molecule ligands such as folate to a DNA reporter strand. This conjugate specifically binds to overexpressed folate receptors on the surface of cancer cells, marking receptor‐positive CTCs. Following removal of unbound conjugates, qPCR is employed to quantify the DNA reporters retained on cell surfaces, enabling ultrasensitive quantification of receptor‐positive CTCs, particularly in NSCLC [155, 156, 157, 158]. Overall, the principal advantage of current PCR‐based phenotypic assays for CTCs lies in their high sensitivity and ability to quantitatively measure protein expression; however, the number of targetable proteins remains limited and requires further expansion.

While phenotypic profiling provides a “snapshot” of current cellular activity, it is inherently limited by CTC heterogeneity. During metastasis, CTCs may exhibit full, partial, or absent expression of key markers. As a result, purely phenotypic analyses risk missing low‐expressing or phenotypically atypical tumor cells. Moreover, most current approaches remain qualitative and restricted in the number of detectable protein targets. Moving forward, future phenotypic detection technologies must focus on enhancing sensitivity and expanding the repertoire of quantifiable proteins. Integration with genotypic and transcriptomic data will be essential to constructing a multidimensional CTC profiling platform—one that captures the complexity of tumor biology and better informs clinical decision‐making.

#### 5.2.4 Combined Detection of Molecular Subtypes Across Different Dimensions

During tumor metastasis, CTCs exhibit extensive phenotypic heterogeneity and distinct gene expression profiles across the EMT and MET states. This heterogeneity evolves over the course of the disease, reflecting the dynamic nature of the tumor [69, 159]. Qualitative characterization based on a single dimension only captures a small fraction of CTC heterogeneity, making it challenging to accurately assess tumor heterogeneity and treatment sensitivity. Increasingly, researchers recognize the importance of comprehensive analysis of the DNA, mRNA, and protein information contained within CTCs [86, 160, 161]. Multidimensional multiplex detection provides more comprehensive data regarding tumor heterogeneity, treatment response, and disease progression, while also accounting for the interactions between different genes and proteins—key for the interpretation of results and clinical applications. As such, integrated molecular and phenotypic analysis of CTCs is emerging as a focal strategy for future liquid biopsy approaches.

As shown in Figure 8A, Wang et al. introduced a novel strategy for reverse enrichment and immunostaining‐fluorescence in situ hybridization (SE‐iFISH) [143]. This method enables the effective simultaneous analysis of surface proteins and nuclear DNA in situ, allowing for the examination of chromosomal aneuploidy and the expression of multiple tumor biomarkers. By analyzing the expression of CEP8 (DNA) along with EpCAM, vimentin, or PD‐L1 (protein), the study provided an integrated classification of CTCs and explored the clinical significance of different CTC subtypes in HCC patients. For the first time, the study demonstrated the presence of small‐sized (≤5 µm) leukocytes with aneuploidy of chromosome 8 in CTCs from HCC patients. Detection of these specific subtypes of CTCs and circulating tumor stem cells (CTSCs) in postoperative patients proved useful for predicting tumor recurrence. This strategy integrates cytogenetic and phenotypic analysis of CTCs, advancing our understanding of cancer metastasis.

**FIGURE 8:**
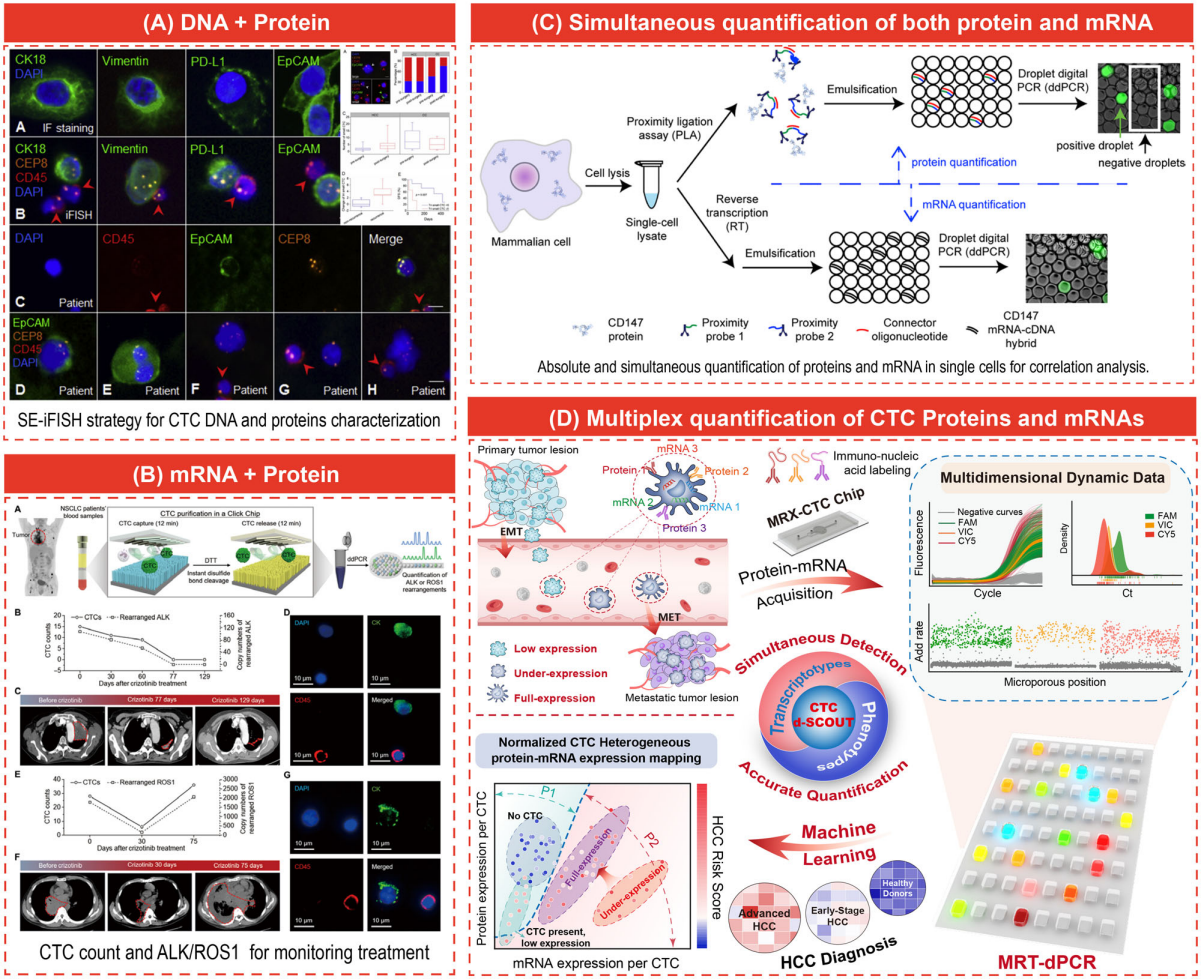
**Methods for integrated multidimensional molecular subtyping detection**. (A) The SE‐iFISH strategy enables phenotypic and karyotypic characterization of CTCs in HCC, providing insights into the clinical significance of various CTC subtypes. Reproduced under the terms of the Creative Commons CC‐BY‐NC‐ND license [143]. Copyright 2017, The Authors. (B) The covalent chemistry‐based nanostructured silicon substrate (Click Chip) facilitates CTC purification and subsequent mRNA analysis, with dynamic changes in CTC count and ROS1 rearrangement copy number offering a means to assess treatment response and disease progression. Reproduced under the terms of the Creative Commons Attribution‐NonCommercial 4.0 International License (CC BY‐NC 4.0) [132]. Copyright 2019 The Authors, some rights reserved; exclusive licensee American Association for the Advancement of Science. (C) Digital Proximity Ligation Assay (dPLA) allows for the absolute quantification of both proteins and mRNA in single mammalian cells. Reproduced with permission [162]. Copyright 2016, Elsevier Inc. (D) The CTC d‐SCOUT method enables synchronized quantification of transcriptional and phenotypic biomarkers for HCC CTCs, greatly enhancing the ability to analyze CTC heterogeneity and accurately quantify the dynamic variations in multidimensional molecular markers during treatment. Reproduced under the terms of the Creative Commons CC BY license [56]. Copyright 2024, The Author(s).

As shown in Figure 8B, Dong et al. made significant progress in combining phenotypic and transcriptomic analyses of CTCs [132]. Using a covalent chemical nanoscale silicon substrate (Click Chip) for capturing and releasing CTCs, this method combines traditional immunofluorescence for labeling and counting with reverse transcription droplet digital PCR (RT‐ddPCR) to quantify copy numbers of oncogene rearrangements, such as ALK/ROS1. In a longitudinal study of NSCLC patients with ROS1 rearrangement, the dynamic changes in CTC count and ROS1 transcript copy numbers correlated with clinical outcomes from radiological imaging. The Click Chip purification process extended the dynamic range for CTC counting and ALK/ROS1 rearrangement quantification, allowing for more precise monitoring of treatment response and disease progression in NSCLC patients.

Furthermore, Albayrak et al. presented an exceptionally sensitive and practical method known as digital proximity ligation assay (PLA, Figure 8C) [162]. This technique employs a pair of DNA‐conjugated antibodies that specifically recognize the target protein. When both antibodies bind in close proximity on the same protein molecule, their attached DNA strands are brought near enough to be joined through a ligation reaction, forming a complete DNA reporter sequence that can be quantitatively detected via digital PCR. This elegant strategy effectively translates the presence of a single protein molecule into an amplifiable and quantifiable DNA signal. Furthermore, by integrating PLA with digital droplet PCR (ddPCR), the authors achieved simultaneous absolute quantification of proteins and mRNA within individual mammalian cells. This study performed copy number counting of endogenous proteins (CD147) and exogenously expressed proteins (GFP‐p65) in hundreds of individual cells, establishing correlations between CD147 mRNA and its encoded protein. This approach led to the construction and validation of a stochastic bi‐stable model, which is crucial for understanding and modeling biological systems with cellular heterogeneity. The high sensitivity and practicality of this method provide a new tool for combined protein and mRNA analysis at the single‐cell level, laying the foundation for deeper insights into cellular heterogeneity and its biological mechanisms.

Recently, Li et al. developed the CTC digital simultaneous cross‐dimensional output and unified tracking (d‐SCOUT) technology (Figure 8D) [56]. This method, based on their proprietary multiple real‐time digital PCR (MRT‐dPCR), enables synchronous quantification of HCC CTC transcriptional and phenotypic biomarkers and establishes a Z‐score risk system closely correlated with clinical staging. By integrating a normalized protein‐mRNA expression profile using machine learning algorithms, the method significantly enhances the ability to analyze CTC heterogeneity and precisely quantify dynamic molecular changes during treatment. This technique overcomes the limitations of traditional single‐dimensional detection, offering a new strategy for multidimensional dynamic monitoring in HCC precision diagnosis and therapy, while also expanding the pathways for studying tumor metastasis mechanisms.

Table 2 summarizes the comparative details of various combined molecular subtype detection techniques. It is evident that in combined molecular and phenotypic analysis of CTCs, due to the distinct biological characteristics of DNA, RNA, and proteins, most researchers employ different techniques or platforms to characterize the various dimensions of molecular information. Typically, due to inconsistent sample requirements, these technologies cannot simultaneously analyze the same sample, and different platforms yield different data outputs. The lack of standardization makes cross‐study comparisons challenging and may lead to discrepancies in data interpretation. While SE‐iFISH presents a simultaneous display of CTC genotype and phenotype information under a unified standard, it still relies on counting, which remains a qualitative approach and has sensitivity limitations [143]. In contrast, dPLA offers a complementary solution, converting cell surface protein information into nucleic acid‐based detection through molecular tagging, then combining it with ddPCR‐based detection of intracellular mRNA, providing multidimensional information under the same standard [162]. However, the PLA technique requires stringent antibody selection and typically needs two antibodies that bind to different epitopes of the target, limiting the number of detectable targets and the breadth of tumor heterogeneity analysis. Moreover, CTC d‐SCOUT [56], by integrating microfluidics and MRT‐PCR, expands the number of molecular dimensions analyzed in tumors and utilizes machine learning for a comprehensive analysis of tumor heterogeneity.

**TABLE 2:** Comparison of combined molecular typing detection technologies at different levels.

Technology name	Target dimension corresponding targets	Detection technique	Same sample source	Simultaneous or sequential presentation	Standardized results	Qualitative/quantitative	Clinical application
SE‐iFISH [143]	DNA: CEP8; protein: EpCAM, Vimentin or PD‐L1	iFISH	Yes	Simultaneous	No	Semi‐quantitative (count)	CTC subtyping, tumor recurrence prediction
AP‐Octopus‐Chip [20]	DNA: KRAS mutation; protein: EpCAM/CK	Sanger Sequencin; IF	No	Sequential	No	DNA: qualitative; protein: semi‐quantitative	Tumor genetic analysis, precise diagnosis
CellSearch and microfluidic RT‐PCR [150]	mRNA: EpCAM and 16 others; protein: EpCAM/CK	RT‐PCR; IF	No	Sequential	No	Semi‐quantitative (Δ Ct difference and count)	Spatial heterogeneity of CTC phenotypes and molecular characteristics
Click Chip [132]	mRNA: ALK/ROS1 rearrangement; protein: EpCAM/CK	RT‐ddPCR; IF	No	Sequential	No	mRNA: Quantitative; Protein: Semi‐quantitative	Evaluate treatment response and disease progression
dPLA technology [162]	mRNA: CD147; protein: CD147	ddPCR	Yes	Simultaneous	Yes	Quantitative	Construct two‐step gene expression model, reveal cellular heterogeneity
CTC d‐SCOUT technology [56]	mRNA: EpCAM, GPC‐3, PD‐L1; protein: EpCAM, GPC‐3, ASGPR	MRT‐dPCR	Yes	Simultaneous	Yes	Quantitative	Heterogeneity analysis; liver cancer diagnosis and treatment efficacy evaluation

Emerging detection paradigms are also accelerating the advancement of multidimensional molecular profiling. Nanozyme‐based biosensors offer a compelling approach by converting protein‐specific binding events into amplified, quantifiable readouts—such as colorimetric or electrochemical signals—that can potentially be integrated with nucleic acid assays within a single microfluidic platform [163, 164, 165]. This synergy between distinct signal transduction mechanisms paves the way for more comprehensive and streamlined cross‐dimensional analyses of individual CTCs.

Integrating multidimensional molecular data allows for a more holistic understanding of the biological properties of CTCs, enhancing the ability to monitor disease progression and refine therapeutic strategies. However, comprehensive molecular and phenotypic profiling of CTCs currently faces two major challenges: first, the absence of a method that can simultaneously capture and preserve both protein and nucleic acid (mRNA or DNA) data from CTCs within a single platform; second, the lack of a standardized approach for the simultaneous quantitative analysis of multidimensional CTC information. Overcoming these challenges will enable more precise utilization of CTCs in cancer research and clinical applications.

#### 5.2.5 PCR for Integrated Multidimensional Molecular Detection

Since its inception, PCR technology has undergone substantial advancements, evolving from basic DNA amplification to real‐time quantitative PCR, digital PCR (chip‐based or droplet‐based), and high‐throughput sequencing. These innovations have profoundly impacted cancer diagnosis, monitoring, and personalized treatment. The inherent sensitivity, specificity, versatility, and integration capacity of PCR technologies have catalyzed the emergence of new techniques, including reverse transcription droplet digital PCR (RT‐ddPCR) [149, 166], droplet digital immuno‐PCR (ddiPCR) [167], LT‐PCR [155, 156, 157, 158], and digital proximity ligation assay PCR (dPLA‐PCR) [162, 168]. These methods are progressively revealing their potential for detecting diverse molecular aspects of tumors, including DNA, RNA, and proteins, as summarized in Table 3.

**TABLE 3:** PCR technologies for detecting cancer information across different dimensions.

Detection technology	Principle	Target dimension	Detection target	Sample source	Performance metrics	Features
Real‐time quantitative PCR [169]	Monitoring allele frequency (AF) changes of ctDNA in 36 cancer‐related genes pre‐ and post‐treatment to assess treatment response	DNA	36 cancer‐related gene mutations (primarily tracking the highest AF variant from baseline)	Plasma	Sensitivity: 99.48% (for SNVs at VAF 0.25%‐0.33%); AF change highly correlates with tumor target lesion change (*R* = 0.66, *p* < 0.001); Response rate: 60.7% in AF decrease group vs 5.8% in increase group (*p* = 0.0003).	Pros: non‐invasive; early prediction of immunotherapy response (by 21 days); dynamic monitoring of disease progression; provides molecular characterization. Cons: dependent on sufficient ctDNA shed by tumor; limited ctDNA from small tumors; high platform cost and requires skilled operators.
Methylation‐specific PCR [170]	Bisulfite treatment of DNA followed by real‐time fluorescent PCR to detect methylation status in specific gene promoter regions	DNA	Methylation of 4 gene promoters (RarB, NISCH, Kif1a, DCC).	Plasma	Lung cancer detection: sensitivity 73%, specificity 71% (AUC = 0.643); non‐/light smokers (<20 pack‐years): sensitivity 72%, specificity 100% (*p* = 0.05); ROC curve with defined methylation thresholds for each gene.	Pros: non‐invasive blood test; capable of distinguishing malignant from non‐malignant CT abnormalities; suitable for high‐risk population screening. Cons: smoking significantly affects methylation background; limited follow‐up data; requires interpretation alongside imaging; detection efficacy for early‐stage lung cancer needs further validation.
RNA‐ddPCR [149]	CTC enrichment via CTC‐iChip (removing hematopoietic cells), followed by whole transcriptome amplification (WTA) and dPCR for liver cancer‐specific RNA	RNA	CTC mRNA (10 transcripts)	Whole blood	Untreated HCC detection: sensitivity 56%; specificity 93%; AUC: 0.80 (LOOCV); mRNA LOD: 52.5 copies/cell.	Pros: not limited by epithelial marker expression; multi‐marker detection improves specificity; suitable for longitudinal monitoring. Cons: requires microfluidic equipment; high RNA quality requirements; complex single‐cell handling techniques.
RT‐ddPCR [166]	Click chemistry‐mediated EV capture and dithiothreitol (DTT)‐mediated EV release, purification of HCC‐specific EVs, detection of signature genes	RNA	Exosomal mRNA (10 transcripts)	Plasma	EV recovery: 81.2%‐94.6%; purity: 85.9%‐99.1%; early HCC detection AUC: 0.93; sensitivity 94.4%, specificity 88.5%.	Pros: specific capture of HCC‐derived EVs; high recovery and purity; digital scoring improves diagnostic accuracy. Cons: complex chip preparation; requires expertise; limited clinical validation samples.
LT‐PCR [155, 156, 157, 158]	Immunomagnetic removal of leukocytes, labeling of folate receptor‐positive CTCs with folate‐oligonucleotide conjugates, washing, and qPCR quantification	Protein	CTC surface folate receptor	Whole blood	Sensitivity: 72.46%/76.37% (training/validation sets); specificity: 88.65%/82.39%; AUC: 0.815/0.813.	Pros: effectively captures EMT phenotype CTCs, not limited by EpCAM expression; higher diagnostic efficacy when combined with traditional tumor markers. Cons: cannot distinguish genetic differences between primary tumors and CTCs; requires further clinical validation.
Triple ddiPCR [167]	Antibody‐DNA conjugates recognize EV surface proteins, triple ddPCR simultaneously detects multiple targets for EV subpopulation counting	Protein	Breast cancer: CD9/CD63/CD81, HER2, EpCAM; Liver cancer: CD9/CD63/CD81, GPC‐3, EpCAM	Plasma	LOD: ∼10 particles/µL; dynamic range: 4 orders of magnitude; breast cancer diagnosis AUC: 0.9427; sensitivity 91.30%, specificity 90.91% (combination of three sEV subpopulations).	Pros: enables single‐vesicle resolution; allows simultaneous detection of multiple subpopulations; features simple operation without complex microfluidic systems; integration with machine learning enhances diagnostic accuracy. Cons: requires validation of antibody specificity; lacks morphological information; dependent on commercial ddPCR platforms.
dPLA [162]	Antibody‐oligonucleotide probes bind the same protein to form an amplifiable DNA template, quantified via ddPCR.	RNA and protein	Protein: CD147, ICAM‐1, GFP; mRNA: CD147	Single‐cell lysate	Protein LOD: 86 molecules/ml ddPCR solution (16.2 fM); sensitivity: ∼70 protein/DNA molecules; dynamic range: 3 orders of magnitude; mRNA recovery: 87.6%.	Pros: absolute quantification of protein and RNA in single cells; high sensitivity; wide dynamic range; simultaneous measurement protein and mRNA from the same cell. Cons: requires specific antibody pairs; requires commercial ddPCR equipment; high technical demand for single‐cell processing.
dPLA [168]	dPLA detects IL‐6 and TNF‐α proteins, ddPCR detects bacterial DNA; combination enables simultaneous host‐pathogen multimarker monitoring	DNA and protein	Proteins: IL‐6, TNF‐α; DNA: Gram‐negative (GN) & Gram‐positive (GP) bacterial 16S rRNA genes; blaTEM antibiotic resistance gene	Bronchoalveolar lavage fluid	IL‐6 LOD: 0.73 fM (0.02 pg/ml); TNF‐α LOD: 4.33 fM (0.08 pg/ml); Bacterial LOD: GN 1.76 CFU/µl, GP 12.19 CFU/µl; blaTEM gene LOD: 1.57 aM.	Pros: ultra‐sensitive (fM level); simultaneous detection of protein and nucleic acid biomarkers; single‐molecule resolution; rapid (results within 2 h); potential for predicting outcomes in septic shock patients. Cons: limited throughput; requires specialized equipment and personnel; high cost.
d‐SCOUT [56]	Combines immuno‐PCR principle (converting CTC protein detection to nucleic acid detection) while preserving mRNA, enabling simultaneous quantification on the same platform	RNA and protein	CTC EpCAM, GPC‐3, PD‐L1 mRNA and EpCAM, GPC‐3, ASGPR proteins	Whole blood	LOD: 3.2 CTCs/mL blood; repeatability (mean %CV): 1.80%–6.05%; diagnostic performance: AUC = 0.950, sensitivity 90.6%, specificity 87.5%.	Pros: simultaneous quantification of protein and mRNA; high sensitivity; enables CTC heterogeneity analysis; supports machine learning clustering; suitable for therapy monitoring. Cons: technically complex, multi‐step process; potentially affected by sample collection/handling; requires special equipment; limited clinical validation samples.

PCR‐based DNA detection enables the identification of gene mutations and methylation changes. For example, in a study involving 206 metastatic colorectal cancer patients, Bettegowda et al. demonstrated that ctDNA analysis for KRAS mutations using quantitative PCR (qPCR) exhibited 87.2% sensitivity and 99.2% specificity [169]. Similarly, in a smaller cohort, qPCR revealed methylation abnormalities in four gene promoters (DCC, Kif1a, NISCH, and Rarb) in plasma from 93 patients with lung abnormalities detected by CT imaging [170]. Seventy‐three percent of cancer patients showed methylation in at least one gene, with a specificity of 71% (*p* = 0.0001) [170].

At the RNA level, PCR, in combination with reverse transcription (RT), has been adapted for the detection of mRNA. Guo et al. developed a negative enrichment‐based qRT‐PCR platform for highly sensitive and reproducible detection of EpCAM mRNA in CTCs, showing significant diagnostic potential in HCC patients, with a 76.7% agreement with CellSearch [171]. Droplet digital PCR (ddPCR), which partitions samples into independent droplet units, enables absolute nucleic acid quantification, offering superior sensitivity and specificity compared to traditional qPCR. To further enhance RNA analysis, researchers have combined RT with ddPCR [132, 149, 166], although the method requires high sample purity. Purification platforms such as iChip [149] for CTCs and Click Chip [166] for exosomes have been developed to address these challenges, enabling high‐throughput, ultra‐sensitive, absolute quantification of target transcripts.

PCR technology also facilitates protein quantification, overcoming the limitations of traditional qualitative CTC protein profiling. Based on immuno‐PCR (iPCR), Lou et al. introduced LT‐PCR, which converts CTC surface ligand information into nucleic acid data [156]. This method, combined with qPCR, successfully detected rare CTCs in NSCLC patients. Liu et al. further integrated iPCR's specificity with ddPCR's sensitivity for multiplex protein quantification on exosome surfaces [167].

Given PCR's excellence in DNA, RNA, and protein detection, it holds great promise for multidimensional molecular analysis under a unified standard. Digital proximity ligation assay (dPLA), which combines iPCR with mRNA cDNA analysis via ddPCR, demonstrates the potential for ultra‐sensitive and absolute quantification of both proteins and mRNA [162, 168]. Abasıyanık et al. applied this method to simultaneously quantify IL‐6 and TNF‐α proteins and 16S rRNA specific to Gram‐negative and Gram‐positive bacteria, predicting mortality and recovery in septic shock with an accuracy exceeding 90% [168]. However, this approach still requires optimization for detecting markers in complex blood samples and needs to expand target coverage to better capture tumor heterogeneity and dynamics.

More recently, real‐time digital PCR (RT‐dPCR) has emerged as a powerful tool for absolute quantification of single molecules. RT‐dPCR enhances the capabilities of traditional dPCR by enabling real‐time monitoring of amplification, offering more precise analysis of low‐abundance proteins or mRNA biomarkers critical for disease diagnosis [172, 173]. This method not only captures endpoint fluorescence but also collects dynamic data from each amplification cycle, facilitating the detection of non‐specific amplifications and enhancing quantitative accuracy [174, 175]. RT‐dPCR, in combination with microfluidic technologies, has enabled highly sensitive, multidimensional molecular quantification of liver cancer CTCs, with a sensitivity of 3.2 CTCs per milliliter of blood [56].

Despite offering an unprecedented lens to dissect CTC heterogeneity, current multimodal molecular analysis platforms still face substantial technical and translational barriers. The major bottlenecks lie in three aspects. First, analyses across molecular layers (DNA, RNA, and proteins) typically rely on distinct, isolated platforms, hindering quantitative integration and causing severe sample loss during multi‐step processing of scarce materials. Second, most combinatorial assays remain technically complex and cost‐prohibitive, limiting their adoption in clinical workflows. Third, prevailing semi‐quantitative or count‐based paradigms fail to capture the continuous dynamics of molecular expression—a critical factor in evaluating functional cellular states. Future technological advances must therefore focus on developing methods capable of simultaneous and absolute quantification of multidimensional biomarkers, while promoting standardized, automated workflows.

Furthermore, despite rapid progress, the clinical translation of CTC detection remains constrained by the dual challenges of poor standardization and insufficient validation. Currently, the de facto “gold standard” in the field—EpCAM‐based immunomagnetic enrichment combined with immunofluorescence identification, exemplified by the **CellSearch** system (the only FDA‐approved CTC detection platform)—provides a unified workflow for enumeration but suffers from intrinsic limitations [176]. Its reliance on EpCAM leads to poor capture of EMT‐transitioned CTC subpopulations, introducing significant biological bias. Likewise, its stringent definition of CTCs as “nucleus‐positive (DAPI^+^), cytokeratin‐positive (CK^+^), and CD45‐negative” may exclude atypical or morphologically distinct cells [177]. Beyond this system, a proliferation of novel technologies has resulted in a fragmented landscape: pre‐analytical variables (e.g., blood collection tube type, preservation time and temperature), enrichment methods (marker‐dependent vs. label‐free), and identification criteria (cell morphology, marker combinations) all vary widely, precluding meaningful cross‐study comparison. This lack of standardization severely hampers clinical validation. Most current studies remain single‐center, small‐cohort, and retrospective, with limited prospective or multicenter trials to substantiate clinical utility. Moreover, standardized protocols linking CTC assay results to actionable clinical decisions—such as therapy selection or real‐time treatment adjustment—are still absent. Therefore, the next decisive step for the field is not only technological innovation, but also the establishment of consensus‐driven technical standards and clinical validation frameworks, enabling a paradigm shift from technology‐driven detection to clinically guided decision‐making.

## 6 AI for Multidimensional Analyses of CTCs

With advances in the enrichment, release, and molecular profiling of CTCs, researchers are now generating vast, high‐dimensional datasets encompassing genomic, transcriptomic, and proteomic information from single cells and CTC clusters. The complexity and heterogeneity of these datasets have quickly surpassed the analytical capacity of conventional bioinformatics tools. AI—particularly machine learning and deep learning—has emerged as a transformative approach to decode the biological significance of CTCs, leveraging its strengths in pattern recognition, nonlinear modeling, and dimensionality reduction [178, 179].

In the context of CTC detection and identification, AI has already demonstrated substantial advantages. For instance, Husseini‐Wüsthoff et al. developed a hybrid machine learning framework combining clustering with a human‐in‐the‐loop strategy to automate CTC detection in blood samples from patients with metastatic breast cancer [180]. By integrating self‐supervised deep learning with adaptable traditional classifiers, and iteratively refining model predictions through expert‐guided labeling of uncertain clusters in latent space, this approach improved classification performance while dramatically reducing manual image review time [180]. Similarly, to tackle the pronounced morphological heterogeneity of CTCs, Wang et al. trained a machine learning model using 9,692 CTC images and over 25 million white blood cell images from 1,703 patients with various cancers—including liver, gastric, bile duct, colorectal, lung, and breast cancer [181]. The model uncovered associations between CTC morphology and clinical outcomes, providing a marker‐independent strategy for CTC identification [181].

Beyond detection, AI‐driven multi‐omics integration (Figure 9) is redefining how CTC biology is understood. Zhang et al. emphasized that applying cutting‐edge multi‐omics technologies to CTCs enables deep dissection of cancer metastasis mechanisms [101]. By jointly analyzing genomic, transcriptomic, and proteomic data, comprehensive molecular landscapes can be constructed, from which AI algorithms identify key drivers of metastasis and therapeutic resistance. In HCC, Zhou et al. developed d‐SCOUT system, which simultaneously quantifies protein markers and mRNA transcripts in CTCs [56]. Machine learning–based clustering of these features generated molecular signatures that accurately reflected metastatic potential and therapeutic response, demonstrating strong diagnostic utility in clinical samples. Such AI‐enabled multidimensional analyses provide powerful tools for decoding tumor heterogeneity and dynamically monitoring treatment efficacy.

**FIGURE 9:**
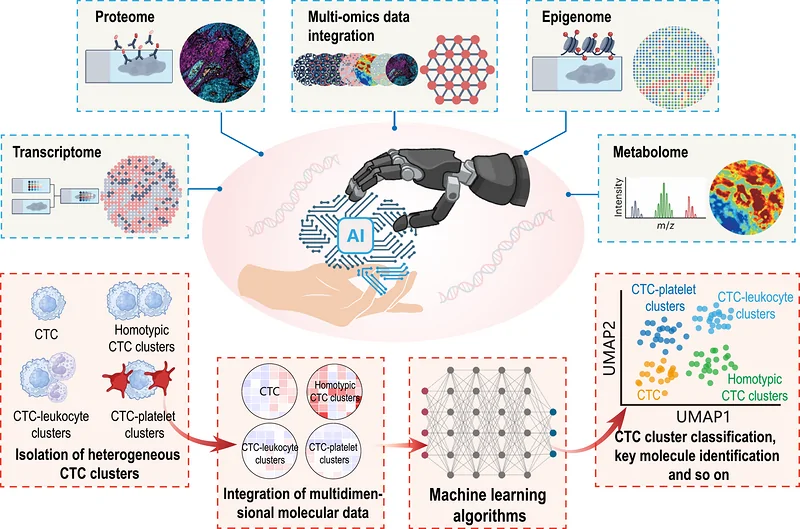
**Application of AI in multimodal analysis of heterogeneous CTC clusters**. AI‐driven frameworks address the challenges of multimodal data heterogeneity. The workflow involves (i) data acquisition from CTCs across genomic, transcriptomic, and proteomic dimensions; (ii) feature extraction and selection using machine learning algorithms to identify the most informative variables; (iii) multimodal data fusion through adaptive weighting strategies to construct integrated molecular interaction networks; and (iv) model output for tasks such as CTC cluster subtyping, prediction of metastatic potential, and inference of key driver molecules. This integrative approach enables a systematic analysis of CTC clusters and supports the development of targeted therapies. Adapted with permission.[183] Copyright 2023, Springer Nature America, Inc.

Detecting heterogeneous CTC clusters—aggregates of tumor cells with divergent phenotypes (for example, epithelial and mesenchymal)—poses distinct methodological demands (Figure 9). Models must capture spatial relationships and intra‐cluster heterogeneity. The aforementioned human‐in‐the‐loop framework, combined with clustering, helps identify and categorize such rare populations [182]. Moreover, unsupervised embedding and clustering applied to single‐cell multi‐omics—using methods such as t‐SNE and UMAP—can reveal subpopulations with distinct molecular programs, illuminating the internal heterogeneity and potential origins of CTC clusters.

Despite these advances, the deployment of AI in CTC research faces several critical limitations. The performance of AI models is heavily dependent on large, high‐quality, and accurately annotated datasets—resources that remain scarce due to the rarity and high cost of generating multi‐omics and clinically linked CTC data. Moreover, the “black‐box” nature of deep learning models limits interpretability, impeding clinical adoption by reducing physician trust in algorithmic predictions. Finally, data standardization remains a pressing issue: significant batch effects and technical discrepancies across laboratories and platforms often cause models trained on one dataset to perform poorly on another. Overcoming these challenges—through robust, generalizable models, interpretable AI frameworks, and standardized data acquisition pipelines—will be essential for realizing the full translational potential of AI in CTC research and precision oncology.

## 7 Conclusion and Perspectives

The highly metastatic nature of cancer significantly complicates clinical decision‐making. Although advances in imaging and molecular diagnostics have expanded our understanding of cancer biology, conventional approaches remain limited by suboptimal sensitivity, poor temporal resolution, and insufficient multidimensional molecular readouts—challenges that continue to hinder the progress of precision oncology. Liquid biopsy, particularly through the analysis of CTCs, offers a minimally invasive window into tumor heterogeneity, therapeutic response, and metastatic potential by enabling the dynamic profiling of DNA, RNA, and protein markers. However, CTC detection and analysis are still confronted with formidable obstacles. Immunoaffinity‐based enrichment methods, which rely on surface antigens such as EpCAM, frequently miss mesenchymal or marker‐negative subpopulations that have undergone EMT. Furthermore, current identification methods are largely restricted to single‐omic measurements, impeding standardization and integration across molecular layers, and limiting clinical translation.

Against this backdrop, material‐driven innovations are emerging as a powerful lever to overcome technical bottlenecks. Label‐free microfluidic technologies—exemplified by functional membrane filtration systems—leverage material properties such as pore size, surface topology, and wettability to physically sort heterogeneous CTC populations. Gradient‐pore polymer membranes can differentiate cells based on mechanical properties, while anti‐adhesive surface coatings reduce non‐specific binding and enhance purity. Nevertheless, current materials still require improvement in biocompatibility, mechanical robustness, and compatibility with downstream multi‐omic analysis. The introduction of smart stimuli‐responsive materials (e.g., thermo‐/photo‐sensitive hydrogels) and bioinspired topographical nanostructures offers new avenues for reversible, non‐destructive CTC isolation at the single‐cell level—providing viable samples for high‐content molecular interrogation.

The integration of microfluidics and functional materials has opened new directions in CTC molecular profiling. Devices engineered from nanostructured materials have enabled the acquisition of high‐fidelity, multidimensional molecular data from CTCs. Digital real‐time PCR, employing high‐throughput microchambers, has achieved unprecedented sensitivity by extracting kinetic parameters from amplification curves in real time. By employing feedback‐optimized reaction conditions and algorithm‐guided curve screening, the system filters out aberrant events, significantly improving the quantification of rare variants such as low‐frequency mutations, fusion genes, and protein markers.

A central challenge that remains is the effective interpretation of multidimensional molecular information. AI, with its capacity for modeling complex, non‐linear relationships, is uniquely positioned to integrate genomic, transcriptomic, and proteomic data, enabling a systems‐level understanding of CTC clonal evolution and microenvironmental interactions [183, 184]. AI‐driven multimodal learning frameworks apply adaptive weighting and feature fusion strategies to construct dynamic molecular interaction networks. This closed‐loop system—integrating materials, detection technologies, and AI—facilitates precise molecular subtyping and real‐time monitoring of CTC clusters, while uncovering the key molecular drivers of their formation through cross‐scale data correlation. Such integration offers a theoretical foundation for developing targeted therapeutics against CTCs and their aggregates.

However, AI is not a panacea. Its depth of application remains constrained by the quality and scale of underlying data. The scarcity of well‐annotated datasets, the opacity of model decision processes, and limited generalizability due to cross‐platform variability all indicate that algorithmic innovation alone has reached its current limits.

Breaking through this bottleneck requires deeper integration between AI and advances in materials and detection technologies. Figure 10 delineates the core trajectory from current challenges to prospective solutions in the field of CTC analysis. Future research should emphasize multidisciplinary convergence: developing multifunctional composite materials that simultaneously enhance CTC enrichment efficiency and multi‐omics compatibility; engineering dynamic, stimuli‐responsive interfaces to enable in situ single‐cell multi‐omics profiling, thereby providing AI models with higher‐quality and more standardized input data. Moreover, by integrating material‐enabled co‐capture of CTCs, exosomes, and ctDNA with AI‐driven multimodal analytics, it will become possible to construct a panoramic atlas of tumor evolution. As intelligent materials, micro/nanofabrication, and single‐cell omics technologies co‐evolve, materials science will drive CTC detection toward greater sensitivity, dimensionality, and dynamism—ultimately positioning it as a cornerstone of precision oncology across the entire cancer care continuum.

**FIGURE 10:**
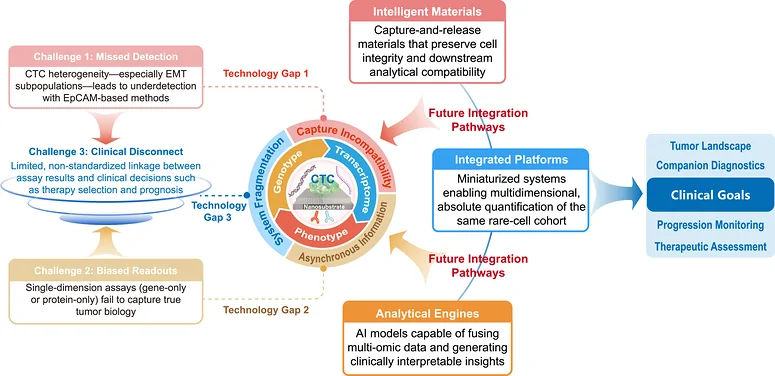
Clinical challenges and future integration pathways in CTC analysis. This figure outlines the conceptual roadmap linking the current challenges of CTC analysis to future integrated solutions. **Left**: Three central clinical barriers are highlighted: (i) under‐detection of CTC subpopulations undergoing epithelial–mesenchymal transition, (ii) biased single‐dimension molecular readouts, and (iii) the disconnect between assay outputs and actionable clinical decisions. **Center**: These issues stem from core technological bottlenecks, including incompatibility between capture strategies and downstream assays, lack of synchronized multidimensional molecular measurements, and fragmentation across analytical steps. **Right**: A forward‐looking framework is proposed, centered on the deep integration of *materials, detection technologies, and AI*. Smart stimuli‐responsive materials enable gentle and lossless cellular manipulation; integrated platforms allow synchronous, absolute quantification of multi‐omic markers; and AI‐driven multimodal analytics construct dynamic molecular interaction networks. Together, these advances form a closed‐loop technological ecosystem poised to transition CTC analysis from experimental methodology to a clinically actionable tool for full‐cycle precision cancer management.

## Author Contributions

**Hao Li** was responsible for the overall structure of the review, literature integration, visualization of figures and tables, and drafting the initial manuscript, contributing primarily to the core sections. **Jiaojiao Sun**, **Kunguo Liu**, **Shijie Wang** and **Bowen Fu** participated in literature screening, case analysis, and figure preparation, assisting with data visualization and the writing of selected sections. **Hong Zhu**, **Peng Cao**, and **Xin Han** provided funding support, guided the research direction and theoretical framework, reviewed the manuscript for logical consistency and academic standards, and facilitated interdisciplinary collaboration.

## Conflicts of Interest

The authors declare no conflicts of interest.

## Data Availability

Data sharing not applicable to this article as no datasets were generated or analysed during the current study
